# Molecular Recognition between Cadherins Studied by a Coarse-Grained Model Interacting with a Coevolutionary Potential

**DOI:** 10.1021/acs.jpcb.0c01671

**Published:** 2020-04-27

**Authors:** Sara Terzoli, Guido Tiana

**Affiliations:** Department of Physics and Center for Complexity and Biosystems, Universitá degli Studi di Milano and INFN, via Celoria 16, Milano 20133, Italy

## Abstract

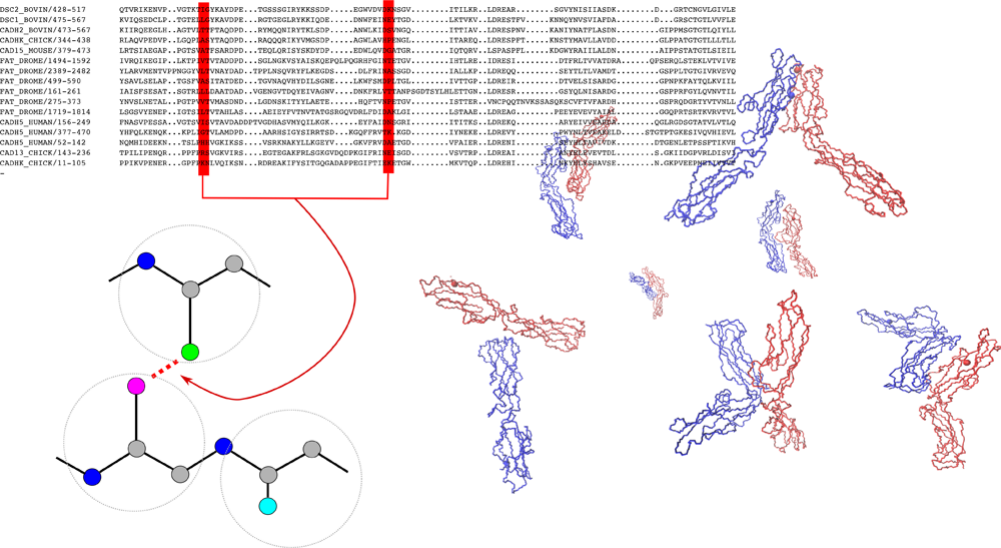

Studying the conformations involved in the dimerization of cadherins is highly relevant to understand the development of tissues and its failure, which is associated with tumors and metastases. Experimental techniques, like X-ray crystallography, can usually report only the most stable conformations, missing minority states that could nonetheless be important for the recognition mechanism. Computer simulations could be a valid complement to the experimental approach. However, standard all-atom protein models in explicit solvent are computationally too demanding to search thoroughly the conformational space of multiple chains composed of several hundreds of amino acids. To reach this goal, we resorted to a coarse-grained model in implicit solvent. The standard problem with this kind of model is to find a realistic potential to describe its interactions. We used coevolutionary information from cadherin alignments, corrected by a statistical potential, to build an interaction potential, which is agnostic about the experimental conformations of the protein. Using this model, we explored the conformational space of multichain systems and validated the results comparing with experimental data. We identified dimeric conformations that are sequence specific and that can be useful to rationalize the mechanism of recognition between cadherins.

## Introduction

Cadherins are surface proteins responsible for cell–cell recognition and adhesion.^1^ They are involved in different stages of tumor progression, like in angiogenesis^2^ and metastasis,^3^ and several germline mutations are found in solid tumors.^4^ For this reason, they are important potential targets of antitumoral molecules.

A large number of members of the cadherin superfamily have been discovered. Particularly important for their relationship with cancer are the so-called “classical” cadherins of types I and II, which are present only in vertebrates and classified according to the tissue where they were first identified. The human genome encodes 114 cadherins for example E-cadherin was found in epithelial tissues, N-cadherin in neurons, and P-cadherin in placenta.^5^

Classical cadherins display five extracellular (EC) domains, which are structurally similar and display a significant sequence similarity, both comparing domains of the same protein type and across different types (see Figure S1 in the Supporting Information). At the interface between consecutive domains, it is bound a calcium ion; the EC domains in the absence of calcium are more flexible^6^ and the protein loses its adhesive function.^7^

The adhesion between two cells is stabilized by the *trans* dimerization of the most distal EC1 domains; crystallization experiments indicate that *trans* dimerization occurs through the swap of their N-termini.^8^ Crystal structures of EC1-EC2 domains, mutated at the N-termini to prevent domain swapping, show that another, X-shaped, *trans* dimeric conformation is possible. Destabilization of the X-dimer by mutating a residue at the junction between EC1 and EC2 slows down the domain-swapping event, qualifying the X-dimer as an on-pathway intermediate.^9^

In many in vivo conditions, classical cadherins are homophilic, in the sense that cells expressing the same cadherin associate, while those expressing different cadherins segregate.^10^ This property is at the basis of cellular binding specificity in tissues and is critical in the correct development of organisms, as ectopic expression of cadherins leads to morphological defects.^11^ However, the homophilic effect in vivo does not seem to be a straightforward consequence of the affinity between cadherins of the same type. In fact, analytical-ultracentrifugation and surface-plasmon-resonance experiments of purified EC1-EC2 domains do not provide dissociation constants that reflect the homophilic relations observed in vivo*.*^12,13^ Although some physical models have been proposed to explain homophilic interactions in systems composed of two cellular types expressing different cadherins,^14,15^ they cannot explain all aspects of cellular sorting^16,17^ and cannot be easily extended to the case of many cell types. Thus, the molecular binding code remains poorly understood, and indeed, it requires further investigation.^18^

Computational methods could in principle complement the available experimental data giving an atomic-level description, analogously to what crystal structures do but also describing the conformational changes and the fluctuations among multiple states associated with the molecular recognition between cadherins. The main problem in this respect is that the system one wishes to simulate, for example that composed of two pairs of EC1-EC2 domains, has a molecular weight of ∼50 kDa, and thus, it is huge from the point of view of standard atomistic simulations in explicit solvent.

Coarse-grained models based on experimental data can be useful in this context. By describing the protein system in implicit solvent and giving a united-atom representation of some atomic groups, they allow computers to sample reasonably fast the conformational space of the system. For example, a C_α_ model interacting with a structure-based potential was used to predict the dimerization constants both for membrane-bound and freely diffusing cadherins.^19^ Similarly, with a simple coarse-grained model, it was possible to simulate the cooperativity between cis and trans interactions.^20^

Defining an interaction potential based on experimental data within the framework of the principle of the maximum entropy, guarantees the realism of the model and minimizes the risk of introducing subjective bias in the description of the system.^21^

In the present work, we employed a coevolutionary interaction potential,^22^ calculated from the set of homologous sequences of the cadherin superfamily. In brief, a coevolutionary potential describes the interaction between amino acids in a protein in such a way to predict the correct correlations between mutations in the alignment of homologs, as obtained from the Pfam database.^23^ This kind of modelling has proven efficient in predicting the native conformation protein monomers^22^ and dimers^24^ of their conformational fluctuations,^25,26^ to study protein aggregation,^27,28^ and the effect of mutations in protein stability.^29,30^ Importantly, we used the coevolutionary potential as it is, without filtering in any way and not including the knowledge of the native structure of the proteins.

We first showed that the model is able to reproduce several experimental data observed for cadherins of different types. Then, we sampled the conformational space of pairs of cadherins of the same kind and different kinds to identify the sequence-dependent dimeric conformations that could be relevant for the mechanism of molecular recognition.

## Methods

Protein chains were modelled with a united-atom representation in an implicit solvent, similar to others commonly employed in the literature.^31,32^ Each amino acid is described^33^ by the positions of its N, CA, and C atoms and by that of another bead, which represents the whole side chain and is set in the position of its center of mass (see Figure S2 in the Supporting Information). Bond lengths and angles are maintained fixed at the values defined by the initial conformation.

The interaction energy of the system is defined as
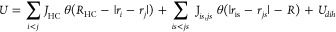
1where *i* and *j* run on all atoms, the function θ(*x*) is a step contact function, which takes the values 1 if *x* ≥ 0 and 0 if *x*<0, *R*_HC_ is the hard-core radius, the hard-core energy *J*_HC_→+ ∞, *is*, and *js* run on the side-chain atoms, *J*_*is*, *js*_ is the interaction matrix, and *R* is the interaction range. We set *R* = 8.5*Å* and *R*_HC_ = 2*Å* for all atoms. The chains are put in a cubic box with hard walls of volume *V*.

The interaction matrix was obtained from a coevolutionary model corrected by a statistical potential. The coevolutionary model takes the alignment of homologs and returns a tensor *J*_*I*, *K*_(σ, τ) of interaction energies between any residue of type σ at position *I* and any residue of type τ at position *K*. This is calculated within the pseudolikelihood approximation^34^ using the code described in ref (35) (see also section S1 in the Supporting Information). Specifically, we obtained the alignment (code PF00028) from the Pfam database,^23^ projecting the alignment onto the sequence of each pdb structure (i.e., removing the sites that correspond to gaps in the sequence of the pdb protein) and discarding those with similarity larger than 90%. In this way, we constructed an alignment of 8 × 10^3^ sequences.

In the standard procedure,^36^ the pseudolikelihood is maximized under the constraint of a *l*_2_ regularizer of kind α∑*_ij_*(*J*_*I*, *K*_(σ, τ))^2^ meant to correct finite-size effects. An important ingredient of the present model is the use of a different type of *l*_2_ regularizer, namely,
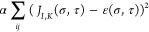
2where ϵ(σ, τ) is a statistical potential^37^ obtained from the frequency of contacts in known protein structures. This is a system-independent potential calculated as
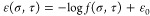
3where *f*(σ, τ) is the frequency of contacts between the side chains of the nonredundant set of proteins of the pdb (with a nonredundancy threshold of 10^–7^). The goal of this regularizer is to provide a priori knowledge of the interactions in the system, especially useful in the case of amino acids that appear with poor statistics or that coevolve under the effect of biological constraints other than the stability of the protein, the effect that would produce falsely strongly interacting pairs.^38^ The regularizer also avoids the problem of choosing a guage for the maximum pseudo likelihood problem.

After calculating the tensor *J*_*i*, *j*_(σ, τ) of interacting energies between any possible pair residue σ and τ that can appear at sites *i* and *j* of the protein, we projected it onto the sequence of interest {σ*_i_*}, obtaining the matrix *J*_*i*, *j*_(σ*_i_*, σ*_j_*). This is then normalized by  to set the scale of two-body interactions to 1. At the variance with the approach of refs (25) and (27), no filtering is applied to the energy elements, any pair of contacts interacting with the corresponding matrix elements is in contact.

The *trans* interaction between identical proteins is set using the same parameters of the corresponding *cis* interaction.

To simulate the rigidifying effect of the Ca^+^ ion at the interface between the EC1 and EC2 domains of cadherins, we implemented an infinite energy well between residues D67 and E101, between E70 and D134, and between R68 and I139.

The last term in the potential of eq 1 depends on the dihedrals of the backbone of the protein, in the form
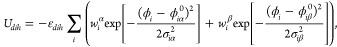
4where ϕ*_i_* are the Ramachandran dihedrals (i.e., alternatively φ and ψ), *w*_*i*_^α^ and *w*_*i*_^β^ are the sequence-dependent propensities of being in α and β structures, respectively, as predicted by PsiPred,^39^ ϕ_*i*α_^0^ and ϕ_*i*β_^0^ are the typical dihedrals associated with α (−63° for φ and −44° for ψ) and β (−105° for φ and −140° for ψ) structures, and we set σ_*i*α_ = 30° and σ_*i*β_ = 40°.

Thus, the potential depends on three energy (meta)parameters, namely, α, ε_0_, and ε*_dih_*. We explored the space of parameters in the case of small proteins (see section S2 in the Supporting Information) and found the optimal values α = 10^–5^, ε_0_ = −1, and ε*_dih_* = 90.

Simulations were performed with a Metropolis Monte Carlo (MC) algorithm, using as elementary moves multiple flips, pivots, and roto-translations of the center of mass of connected systems of chains.^40^ To generate the initial conformations for the simulations, we started from the pdb structure, mapped onto the coarse-grained model (i.e., removing O, N, and H atoms and placing the sidechan bead at the center of mass of the sidechain); in simulations of the dimer, we place the two chains randomly in the box. Then a low-temperature simulation (*T* = 10^–3^) is carried out mainly to remove the steric clashes associated with the potential defined by eq 1. Parallel-tempering simulations^41^ were performed by trying an exchange between replicas of adjacent temperatures every 1000 MC steps.

Since protein domains tend to attract each other quite strongly, we performed an umbrella sampling^42^ to equilibrate the system more efficiently. The interaction between different chains is rescaled by a factor *k <* 1*.* The correct equilibrium probabilities *p*(*r*) of the conformations of the system are then recovered a posteriori from the simulated probabilities *p_k_*(*r*) as
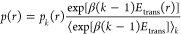
5where *E*_trans_(*r*) is the rescaled interaction between the chains, β = 1/*T* is the inverse temperature (we set Boltzmann’s constant to 1, expressing temperatures in energy units), and the angular brackets with the subscript *k* at the denominator indicate the average obtained by the simulation. In this way, we could make the simulation faster, having a larger *k*_*of*f_ between the two chains, and recover the correct equilibrium properties a posteriori.

From the simulations of the dimer with rescaled trans interactions, we calculated the dissociation constants as
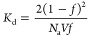
6where *f* is the fraction of dimeric conformations, obtained from the simulation after rescaling with eq 5, *N*_a_ is Avogadro’s number, and *V* is the volume of the box containing two molecules. A conformation is defined dimeric if there is any *trans* attractive contact between any two atoms of the two chains.

## Results

### Coevolutionary Potentials Reproduce the Native State of Monomeric EC1

**Figure 1:**
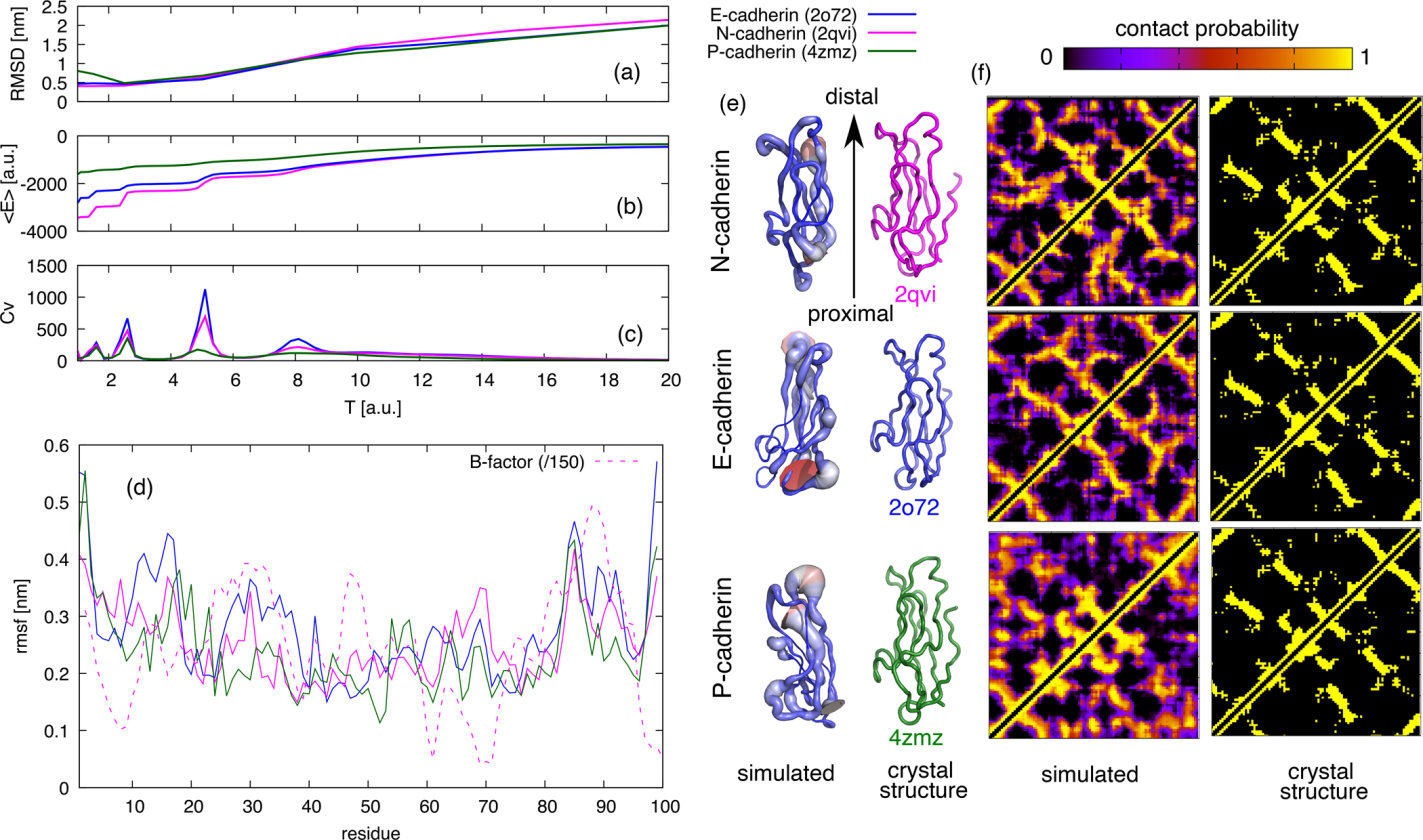
Simulations of monomeric EC1 domains of N-, E-, and P-cadherins. (a) The average RMSD to the crystallographic structures, (b) the average energy, (c) and the specific heat with respect to simulation temperature (in energy units) are displayed for the three monomers. (d) The conformational fluctuations of the simulated proteins calculated at *T* = 3.6, compared to the b-factors (dashed line) of the monomeric N-cadherin 1NCJ. (e) The equilibrium structures obtained from the simulations in which the thickness of the cartoon reflects the fluctuations of the corresponding monomers (left side), compared to the crystallographic structures (right side). (f) The contact probabilities obtained from the simulations at *T* = 3.6, compared to the crystallographic contact maps.

As a first step, we simulated the dynamics of EC1 of N-, E-, and P-cadherins (residues 1–99) in conditions of infinite dilution where the protein is monomeric. We used as putative monomeric reference conformations the crystallographic structures 2qvi (for N-cadherin), 2o72 (for E-cadherin), and 4zmz (for P-cadherin). At low temperature, the average RMSD calculated from parallel-tempering simulations with respect to the crystallographic conformations is ≈0.5 nm for N- and E-cadherins and ≈0.7 nm for P-cadherin (see Figure 1a). This is comparable with that of the proteins previously studied (cf. section S2 in the Supporting Information). The calculated contact-probability maps are also native-like (cf. Figure 1f). These two facts suggest that the minimum requirement for the model to be useful that is to have the experimental conformations as low-temperature equilibrium states is met. It is important to stress that, unlike structure-based models,^43^ here, the model is agnostic of the native conformation of the proteins. Moreover, the use of the regularizer given by eq 2 based on a system-independent statistical potential seems important to obtain a realistic potential since not using it increases markedly the RMSD of the protein (cf. Figure S3).

At varying temperature, all three proteins display a main transition at temperature *T* ≈ 5 (in energy units, see Figure 1c) between a native and denatured state. The transition, as described by the model, appears as poorly cooperative; although we are not aware of calorimetric studies of the EC1 domains of cadherins, it is likely that, as in most implicit-solvent models,^44^ this is an artifact associated with the use of reduced degrees of freedom.

The thermal fluctuations of the residues display similar patterns in the three monomeric cadherins (see Figure 1d), but their relative widths are protein-dependent (see Figure 1e): E-cadherin displays larger fluctuations in the proximal region (i.e., that linked to EC2 in the full complex), while P-cadherin fluctuates more in the distal region, and N-cadherin behaves in an intermediate way (cf. Figure 1e). The fluctuations of the residues of N-cadherin display a significant correlation (Pearson’s *r* = 0.47, p-value <10^–5^) with the b-factors of its crystallographic structure (see dashed line in Figure 1d; note that 1NCJ is the only available structure of a monomeric EC1 domain of a classical cadherin).

### Model Correctly Predicts the Dimeric Structures of EC1 of N-Cadherin

**Figure 2:**
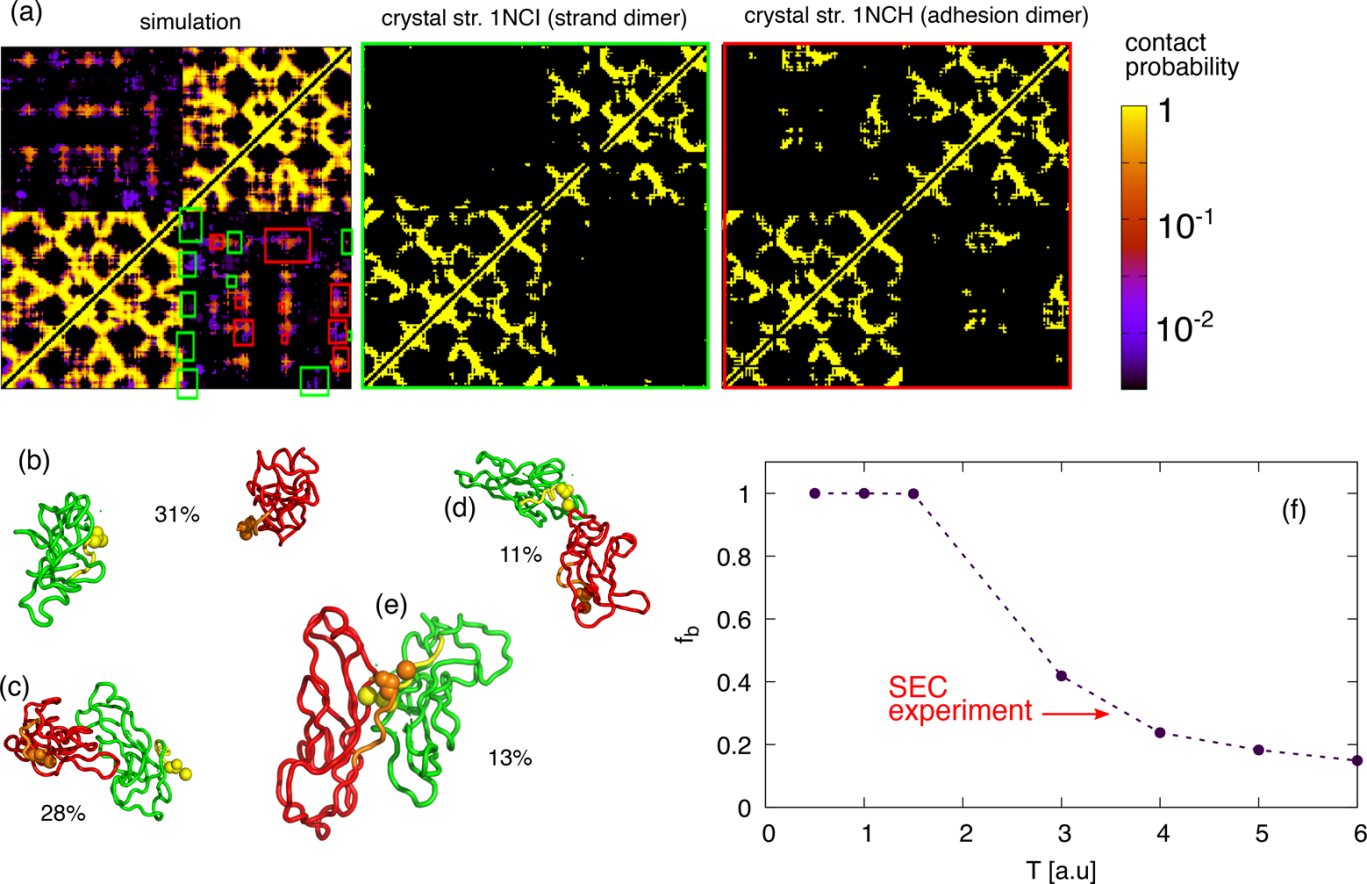
Result of simulations of two EC1 domains of N-cadherin at *T* = 3.6. (a) The calculated average contact map compared with that of crystal structures 1NCI and 1NCH. The colored boxes highlight the intrachain contacts present in the crystal structures (1NCI in green and 1NCH in red). Some representative structures obtained from a clustering of the trajectories are (b) the two disjoint chains, (c) a conformation resembling the adhesion dimer, (d) a conformation bound at the ends, (c) and a conformation resembling the domain-swapped strand dimer. The associated percentages indicate the fraction of the trajectory in each cluster. (f) The fraction *f*_b_ of bound monomers as a function of temperature. The red arrow indicates the experimental value.

The next step was to simulate two EC1 domains, which is the system studied in the original work of Shapiro and coworkers.^8^ The two chains are put in a spherical box of volume *V* ≈ 3.2 × 10^4^ nm^3^, corresponding to a concentration of ≈100 μM. The fraction *f*_B_ of conformations displaying inter-chain contacts is displayed in Figure 2f. The experimental *k*_D_ obtained at room temperature from analytical size-exclusion chromatography on EC1 alone is 166 μM,^45^ corresponding to a *f*_B_*=* 0.3 in the simulation volume. This allowed us to set the simulation temperature *T* = 3.6 (in energy units) as that corresponding to room temperature (see red arrow in Figure 2f).

The average contact map simulated at *T* = 3.6 is displayed in Figure 2a, together with the contact maps of the two alternative crystallographic structures found for EC1^8^ describing the domain-swapped “strand dimer” (pdb code 1NCI) and the “adhesion dimer” (pdb code 1NCH). The binding of the two monomers does not perturb their internal structure, the intrachain contacts remain the same as the crystallographic ones, and the average RMSD remains ≈0.5 as in the monomeric case (cf. Figure 1a).

The simulations also display several interchain contacts of varying stabilities. Two sets of contacts, marked with green and red boxes in Figure 2a, correspond to the contacts of the strand and adhesion dimers, respectively. Another set of contacts, formed with nonnegligible probability, cannot be explained by the available crystallographic structures.

The simulated conformations were clustered based on their mutual similarity, and the most representative conformations are shown in Figure 2b–e, together with the associated probabilities. In approximately one third of the conformations, the two chains are disjoint (Figure 2b); in another third, they display a conformation similar to that of the adhesion dimer (Figure 2c), making the interchain contacts marked with red boxes in Figure 2a. In 13% of the sampled conformations, the tryptophans 2W of each chain is in contact with the hydrophobic pocket of the other chain (see Figure 2e and the green-boxed contacts in Figure 2a). An 11% of the conformations populates a dimeric conformation (Figure 2d), which is not similar to any available crystallographic structure, while the remaining 17% of the conformations populates dimeric structures that cannot be easily clustered into well-defined groups.

### Simulated EC1-2 Fluctuate among Different Conformations, Including X- and Domain-Swapped Dimers

**Figure 3:**
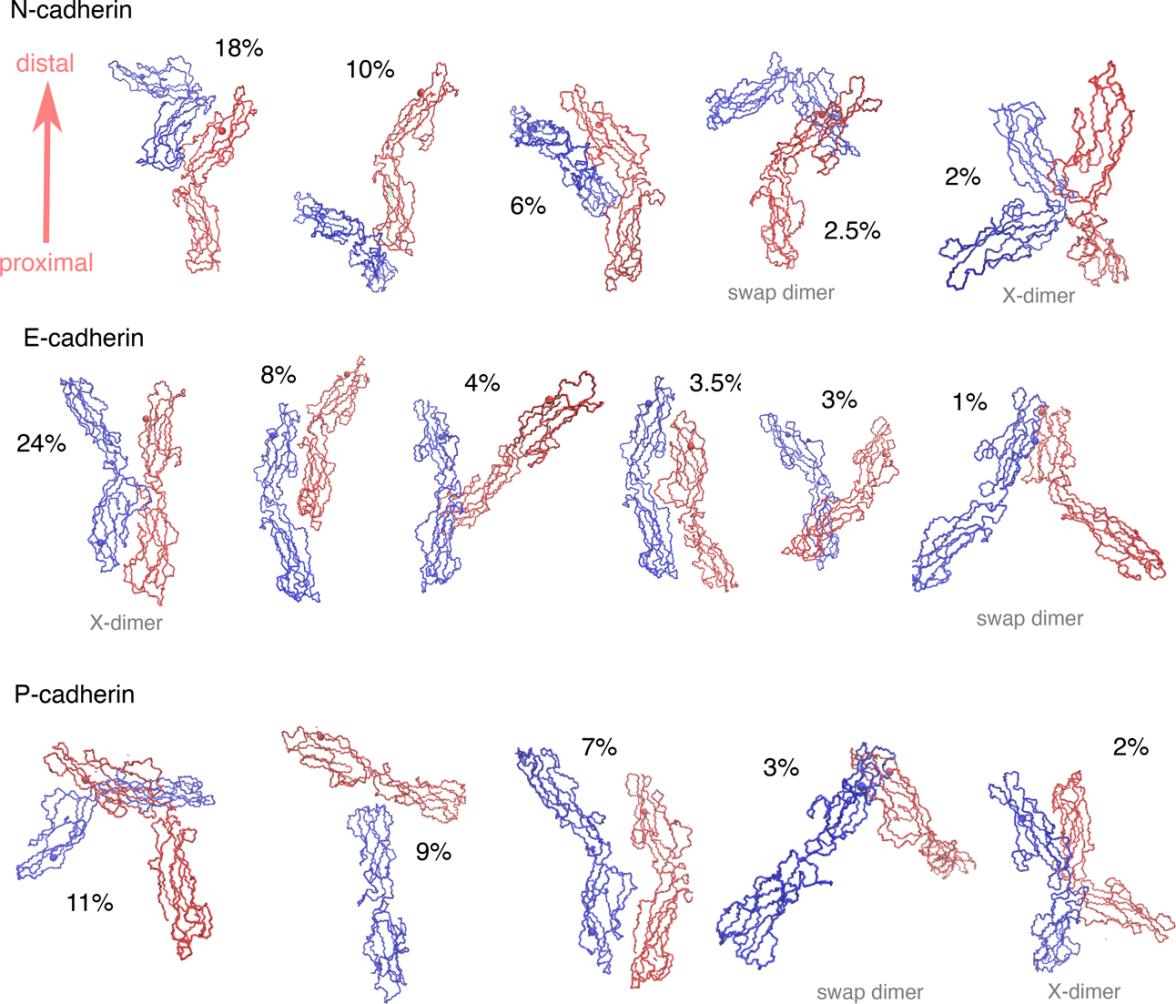
Representative conformations of clusters obtained from the simulation of the EC1-2 dimer of N-, E-, and P-cadherins. For each representative, the percentage of conformations populating that cluster is indicated. The red monomers are oriented to display its distal end upward.

Simulations of two copies of the chain composed of the EC1-2 domain are carried out for 10^9^ MC steps for N-, E-, and P-cadherins, rescaling the interchain interactions as described in the Methods section. All proteins display a very heterogeneous set of dimeric conformations. A clustering analysis whose results are reported in Figure 3 reports that the three proteins can assemble in many possible conformations, and among them, there are conformations resembling the swap-dimer and X-dimer although with a probability lower than expected. In several conformations, the two chains are side-by-side or display interactions between their proximal ends.

**Figure 4:**
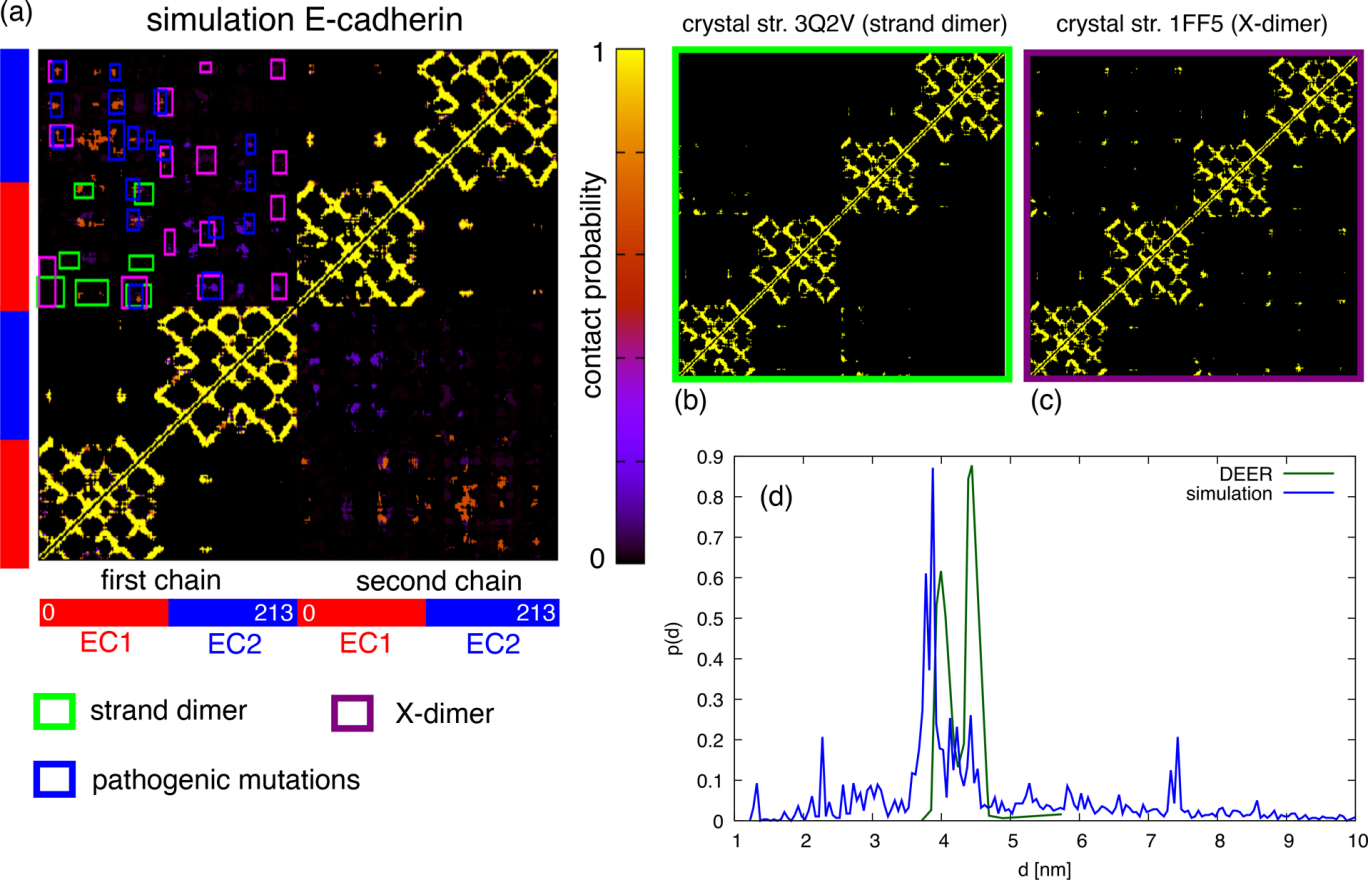
(a) Simulated contact map of E-cadherin; the lower-left and upper-right quadrants display intrachain contacts, the upper-left and lower-right quadrant display contacts between the two chains. (b) As a reference, we displayed the contact maps of the crystal structures of the strand dimer and X-dimer, and their contacts (with probability larger than 0.1) are reported with green and purple boxes, respectively, in the simulated map. The blue boxes indicate contacts between residues whose mutation is observed in tumoral tissues. (d) Comparison between the distribution of distances between residues 73–75 and residues 114–116 calculated from the simulation (in blue) and measured by DEER.

In the case of E-cadherin,^46^ the average contact map is displayed in Figure 4a. Of the 45 clusters of contacts with probability larger than 0.1, which are apparent in the contact map, 7 are those that stabilize the strand dimer (marked with green boxes, cf. also Figure 4b) and 15 are those that stabilize the X-dimer (purple boxes, cf. also Figure 4c). The remaining contacts cannot be explained from the crystallographic structures, but results from other conformations are displayed in Figure 3. However, 18 of these unexplained contacts involve all residues that are known to be associated with mutations observed in tumoral cells that decrease cell–cell adhesion and induce metastasis.^4^

The contact maps simulated for P- and N-cadherins display overall fewer clusters of contacts (cf. Figure S6 in the Supporting Information). N-cadherin has 26 clusters, 6 of them are associated with the strand dimer, 11 with the X-dimer, and 10 with other conformations that are displayed in Figure 3. P-cadherin^47^ has 21 clusters, 5 of them are associated with the strand dimer, 11 with the X-dimer, and 6 with other conformations.

For E-cadherin, one can also compare the results of simulations with those of double electron–electron resonance (DEER), which is able to measure the distribution of distances between labelled side chains within 6 nm. E-cadherin labelled at residues 73–75 and 114–116 display a double peak between 4.0 and 4.5 nm, interpreted as arising from the strand dimer.^13^ Our simulations display a similar double peak (cf. Figure 4d) as the result of the contribution of all conformations and is displayed in Figure 3.

On the other hand, the simulated distances between residues 135 of the two chains match poorly with the results obtained by DEER (cf. Figure S7 in the Supporting Information), most likely because the angle between the axes of the two chains is strongly affected by the coarse graining of the model.

### Simulations of Heterophilic Complexes

**Figure 5:**
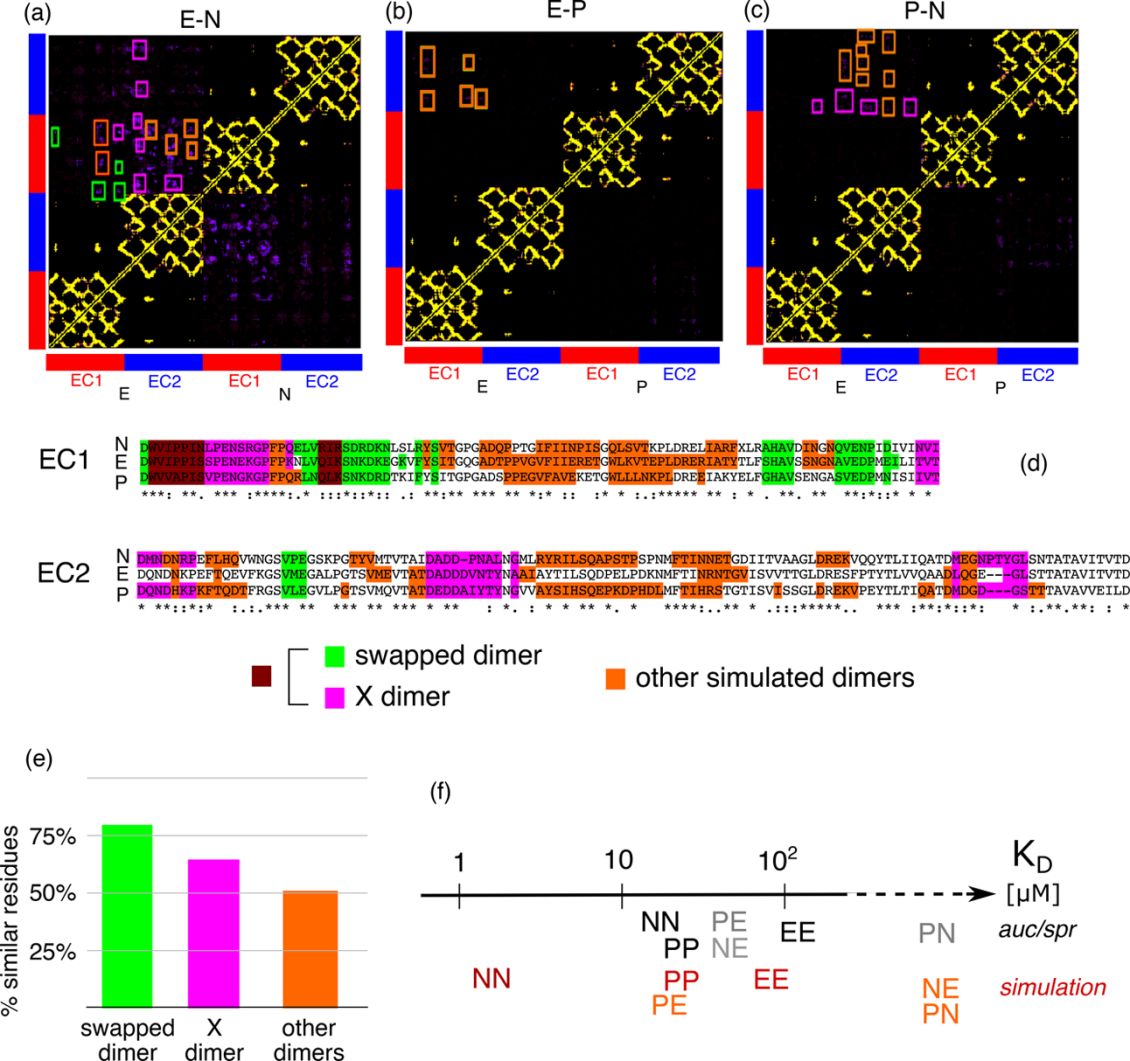
Average contact maps obtained simulating the EC1-2 domains of hybrid systems composed of (a) E- and N-cadherins, (b) E- and P-cadherins, (c) and N- and P-cadherins. The colored boxes indicate the contacts of the swapped dimer (in green), X-dimer (in purple), and other dimeric conformations sampled in the simulation (in orange). (d) A comparison of the sequences of the three cadherins in which identical residues are marked with a star, and chemically similar residues are marked with a colon. (e) The percentage of residues associated with the dimeric structures that are similar in the three proteins. (f) The experimental and simulated dissociation constants (cf. also Table S1) for the various systems under study.

Further simulations were carried out with pairs of different EC1-2 domains to simulate heterophilic interactions. The average contact maps of the hybrid systems are displayed in Figure 5a–c. The system E-N populates in a detectable way the swapped and the X-dimer and the system P-N only the X-dimer, while E-P is none of the two. However, all systems populate multiple dimeric conformations most of which are system dependent.

The residues participating in the dimeric contacts are highlighted in the alignment displayed in Figure 5d. It is apparent that residues participating to the swapped dimer are more conserved than those participating to the X-dimer, and those participating to the other types of dimers are even less conserved (cf. also Figure 5e). As a consequence, the contacts that stabilise these other types of dimers are more specific than those in swapped and X-dimers.

The dissociation constants obtained from the simulations for the homophilic and heterophilic cases respect, in most cases, the order of those obtained experimentally from analytical ultracentrifugation and plasmon resonance analysis,^13^ see Figure 5f. The major difference is in the fact that the N-E complex is very weak (*k*_D_ > 100 μM) in the simulations while should be of the same order of magnitude as the P-E complex (∼50 μM).

## Discussion

The atomic-scale picture that we have of the encounter mechanism between cadherins is essentially based on the crystal structures of the wild-type and of mutant proteins.^8,9,13,46^ The behavior of cadherins in solution, and even more in vivo, could be more complex than the static and homogeneous situation observed in crystals. Unfortunately, for such a large system as an assembly of cadherins, there are a few experimental techniques that can report indirect conformational data in solution,^13,48−51^ leaving behind the problem of turning these data into a structural understanding of their recognition mechanism.

Simulating the mutual search and binding of multiple cadherins with computational techniques can be a way to obtain details that can complement experimental data and describe all the conformations involved in the mechanism of molecular recognition at the atomic level. The main problem in pursuing this approach with standard molecular-dynamics simulations is that one having to deal with a system that on the scale of computer calculations is large (∼50 kDa for the EC1-2 dimer) and takes a long time to bind (∼1 s for E-cadherin^48^).

Coarse-grained models, combined with advanced sampling algorithms, can be useful to study this kind of system. Using a united-atom representation in which each amino acid is represented by 4 atoms and the solvent is treated implicitly, we could sample at equilibrium the conformational space of two copies of the EC1-2 domains with advanced Monte Carlo algorithms in a few days of the computational time. With this model, even the simulation of the whole EC1-5 system and more than two chains, necessary to account for the cooperativity associated with cadherin clustering,^20^ does not appear to be computationally unreachable.

The main problem with computational models of biomolecules and coarse-grained model in particular is to build a realistic interaction potential. A strategy that is gaining popularity is to build potentials based on available experimental data in the framework of the principle of the maximum entropy and then to validate the model with independent data.^52,53^

A particularly abundant set of data available for proteins and cadherins in particular is sequence data in the form of alignments of homologous protein sequences. Coevolutionary analysis is a way to extract a contact potential from these data, finding the most likely potential that could have produced the available alignment as the result of natural evolution. There are several implementations of this idea,^26,34^ all of them giving comparable results.^38^ Coevolutionary potentials proved to be useful in predicting the native state of single-domain proteins,^26,34^ their energy profile,^25^ protein–protein interactions^54^ to study protein aggregation,^27,28^ and the thermodynamic effect of point mutations.^29,30^

In the present work, we applied a coevolutionary potential to the problem of molecular recognition between cadherins. The coevolutionary potential was corrected with a system-independent statistical potential, obtained from the contact probabilities obtained from the whole pdb. This appears to be an important step because it corrects those terms of the coevolutionary potential associated with poor statistics in the cadherin alignments and then otherwise affected by large noise.

We validated the model in several ways, also estimating what are its limitations. First, we verified that the monomeric system displays at low temperature a unique native conformation compatible with the crystallographic one. This was tested for the EC1 domain of three different cadherins and for two other small proteins used as independent control. The positive result that we obtained is not straightforward because, unlike structure-based models widely used to study conformational changes in proteins,^55^ we never used any information about the native conformation of the system during the construction of the potential. The accuracy with which we could simulate the native state of monomers is somewhat worse than the experimental resolution of X-ray structures, being quantified by an RMSD of the order of 0.5–0.7 nm. This is due to the united-atom modelling of amino acids that is required to fasten the simulation but that does not lead to a perfect packing of side chains.

Moreover, we compared the simulated trajectories with the experimental b-factor, with the results of analytical size-exclusion chromatography, with double electron–electron resonance experiments and with the dissociation constants obtained by analytical ultracentrifugation and plasmon resonance analysis. Also, the dimeric structures generated by the simulations were compared to the crystallographic structures available for the N-, E-, and P-cadherins. Interestingly, in all the three simulations, we find structures similar to the swap-dimer andX-dimer that were identified in crystals. Since wild-type cadherins crystallize into swap-dimers, one would have expected that this structure displayed the largest population fraction in the simulation. One reason why this is not the case could be that the swap-dimer is easier to crystallize, and thus, the experiments select only one of the possible conformers. Of course, the two-body terms in the potential are the results of several approximations, and the coarse-graining of the model also affects the entropy of the system. These are quantities on which that the probability depends exponentially and consequently affects consistently the statistical weight of the sampled conformations.

A way to improve the results, matching better the experimental knowledge we have of the system, is to include it in the simulation as an energy term in the framework of the principle of the maximum entropy,^52,56^ as done for an example in ref (28). A drawback of this strategy in the case of cadherins is the scarcity of data at conformational level and thus the impossibility of validating the results with independent data.

One can thus wonder whether the heterogeneous set of binding modes of the EC1-2 dimers observed in the simulations is realistic or just an artifact of the model. An element pointing toward their realism is that all pathogenic mutations observed in E-cadherin^4^ affect the interface of these conformations, leading in vivo to a diminished adhesion and increased migration propensity of the cells. This fact suggests that the dimeric conformers we found may play a role in the overall binding mechanism.

This fact becomes more evident when simulating heterophilic interactions in hybrid systems composed of different types of cadherins. Also in this case, we could observe conformations resembling the swapped and the X-dimer, in addition to a complex set of other dimeric conformations. Interestingly, while the residues that stabilize swapped and X-dimers are quite independent on the kind of cadherin and thus weakly specific, the other dimers interact through contacts that are much more system dependent. One can then speculate that the multiplicity of dimeric conformations different from the swapped and X-dimers play a role in the selective molecular recognition between cadherins.

## Conclusions

We proposed a coarse-grained model interacting through a potential based on the coevolutionary analysis of homologous proteins to study the molecular recognition between molecules that are too large to be studied with standard molecular-dynamics simulations. The model relies on sequence data and is agnostic of the structural properties of the protein. It was validated comparing the results of Monte Carlo simulations with experimental data of various types, giving good agreement except for the relative populations of the different types of dimeric structures that depend exponentially on the energies that define the model, and are thus quite sensitive to them.

A thorough sampling of the conformational space of dimers composed of pairs of EC1-2 domains of E-, P-, and N-cadherins show that, besides the known swapped and X-dimers, the systems populate multiple other dimeric conformations, which are more sequence-dependent and thus could play an important role in the selectivity of molecular recognition between cadherins.

## Supporting Information Available

pseudolikelihood approximation and of the tuning procedure of the model parameters (PDF)

## References

[ref1] Takeichi M. Cadherin Cell Adhesion Receptors as a Morphogenetic Regulator. Science 1991, 251, 1451–1455. 10.1126/science.2006419. 2006419

[ref2] Blaschuk O. W. N-Cadherin Antagonists as Oncology Therapeutics. Philos. Trans. R. Soc. Lond. B. Biol. Sci. 2015, 370, 20140039 10.1098/rstb.2014.0039. 25533096 PMC4275908

[ref3] Berx G.; Becker K. F.; Höfler H.; van Roy F. Mutations of the Human E-Cadherin (CDH1) Gene. Hum. Mutat. 1998, 12, 226–237. 10.1002/(SICI)1098-1004(1998)12:4<226::AID-HUMU2>3.0.CO;2-D. 9744472

[ref4] Petrova Y. I.; Schecterson L.; Gumbiner B. M. Roles for E-Cadherin Cell Surface Regulation in Cancer. Mol. Biol. Cell 2016, 27, 3233–3244. 10.1091/mbc.E16-01-0058. 27582386 PMC5170857

[ref5] Tiwari P.; Mrigwani A.; Kaur H.; Kaila P.; Kumar R.; Guptasarma P. Structural-Mechanical and Biochemical Functions of Classical Cadherins at Cellular Junctions: A Review and Some Hypotheses. Adv. Exp. Med. Biol. 2018, 1112, 107–138. 10.1007/978-981-13-3065-0_9. 30637694

[ref6] Häussinger D.; Ahrens T.; Sass H.-J. J.; Pertz O.; Engel J.; Grzesiek S. Calcium-Dependent Homoassociation of E-Cadherin by NMR Spectroscopy: Changes in Mobility, Conformation and Mapping of Contact Regions. J. Mol. Biol. 2002, 324, 823–839. 10.1016/S0022-2836(02)01137-3. 12460580

[ref7] Sedar A. W.; Forte J. G. Effects of Calcium Depletion on the Junctional Complex between Oxyntic Cells of Gastric Glands. J. Cell Biol. 1964, 22, 173–188. 10.1083/jcb.22.1.173. 14195608 PMC2106493

[ref8] Shapiro L.; Fannon A. M.; Kwong P. D.; Thompson A.; Lehmann M. S.; Grübel G.; Legrand J. F.; Als-Nielsen J.; Colman D. R.; Hendrickson W. A. Structural Basis of Cell-Cell Adhesion by Cadherins. Nature 1995, 374, 327–337. 10.1038/374327a0. 7885471

[ref9] Harrison O. J.; Bahna F.; Katsamba P. S.; Jin X.; Brasch J.; Vendome J.; Ahlsen G.; Carroll K. J.; Price S. R.; Honig B.; et al. Two-Step Adhesive Binding by Classical Cadherins. Nat. Struct. Mol. Biol. 2010, 17, 348–357. 10.1038/nsmb.1784. 20190754 PMC2872554

[ref10] Duguay D.; Foty R. A.; Steinberg M. S. Cadherin-Mediated Cell Adhesion and Tissue Segregation: Qualitative and Quantitative Determinants. Dev. Biol. 2003, 253, 309–323. 10.1016/S0012-1606(02)00016-7. 12645933

[ref11] Detrick R. J.; Dickey D.; Kintner C. R. The Effects of N-Cadherin Misexpression on Morphogenesis in Xenopus Embryos. Neuron 1990, 4, 493–506. 10.1016/0896-6273(90)90108-R. 2322458

[ref12] Katsamba P.; Carroll K.; Ahlsen G.; Bahna F.; Vendome J.; Posy S.; Rajebhosale M.; Price S.; Jessell T. M.; Ben-Shaul A.; et al. Linking Molecular Affinity and Cellular Specificity in Cadherin-Mediated Adhesion. Proc. Natl. Acad. Sci. U. S. A. 2009, 106, 11594–11599. 10.1073/pnas.0905349106. 19553217 PMC2710653

[ref13] Vendome J.; Felsovalyi K.; Song H.; Yang Z.; Jin X.; Brasch J.; Harrison O. J.; Ahlsen G.; Bahna F.; Kaczynska A.; et al. Structural and Energetic Determinants of Adhesive Binding Specificity in Type I Cadherins. Proc. Natl. Acad. Sci. U. S. A. 2014, 111, E4175–E4184. 10.1073/pnas.1416737111. 25253890 PMC4210030

[ref14] Steinberg M. S. Reconstruction of Tissues by Dissociated Cells. Science 1963, 141, 401–408. 10.1126/science.141.3579.401. 13983728

[ref15] Harris A. K. Is Cell Sorting Caused by Differences in the Work of Intercellular Adhesion? A Critique of the Steinberg Hypothesis. J. Theor. Biol. 1976, 61, 267–285. 10.1016/0022-5193(76)90019-9. 985668

[ref16] Pawlizak S.; Fritsch A. W.; Grosser S.; Ahrens D.; Thalheim T.; Riedel S.; Kießling T. R.; Oswald L.; Zink M.; Manning M. L.; et al. Testing the Differential Adhesion Hypothesis across the Epithelial–mesenchymal Transition. New J. Phys. 2015, 17, 083049 10.1088/1367-2630/17/8/083049.

[ref17] Krieg M.; Arboleda-Estudillo Y.; Puech P.-H.; Käfer J.; Graner F.; Müller D. J.; Heisenberg C.-P. Tensile Forces Govern Germ-Layer Organization in Zebrafish. Nat. Cell Biol. 2008, 10, 429–436. 10.1038/ncb1705. 18364700

[ref18] Green J. B. A. Sophistications of Cell Sorting. Nat. Cell Biol. 2008, 10, 375–377. 10.1038/ncb0408-375. 18379595

[ref19] Wu Y.; Honig B.; Ben-Shaul A. Theory and Simulations of Adhesion Receptor Dimerization on Membrane Surfaces. Biophys. J. 2013, 104, 1221–1229. 10.1016/j.bpj.2013.02.009. 23528081 PMC3602765

[ref20] Wu Y.; Jin X.; Harrison O.; Shapiro L.; Honig B. H.; Ben-Shaul A. Cooperativity between Trans and Cis Interactions in Cadherin-Mediated Junction Formation. Proc. Natl. Acad. Sci. 2010, 107, 17592–17597. 10.1073/pnas.1011247107. 20876147 PMC2955114

[ref21] Jaynes E. T. Information Theory and Statistical Mechanics. Phys. Rev. 1957, 106, 620–630. 10.1103/PhysRev.106.620.

[ref22] Morcos F.; Pagnani A.; Lunt B.; Bertolino A.; Marks D. S.; Sander C.; Zecchina R.; Onuchic J. N.; Hwa T.; Weigt M. Direct-Coupling Analysis of Residue Coevolution Captures Native Contacts across Many Protein Families. Proc. Natl. Acad. Sci. U. S. A. 2011, 108, E1293–E1301. 10.1073/pnas.1111471108. 22106262 PMC3241805

[ref23] Finn R. D.; Coggill P.; Eberhardt R. Y.; Eddy S. R.; Mistry J.; Mitchell A. L.; Potter S. C.; Punta M.; Qureshi M.; Sangrador-Vegas A.; et al. The Pfam Protein Families Database: Towards a More Sustainable Future. Nucleic Acids Res. 2016, 44, D279–D285. 10.1093/nar/gkv1344. 26673716 PMC4702930

[ref24] dos Santos R. N.; Morcos F.; Jana B.; Andricopulo A. D.; Onuchic J. N. Dimeric Interactions and Complex Formation Using Direct Coevolutionary Couplings. Sci. Rep. 2015, 5, 13652 10.1038/srep13652. 26338201 PMC4559900

[ref25] Sutto L.; Marsili S.; Valencia A.; Gervasio F. L. From Residue Coevolution to Protein Conformational Ensembles and Functional Dynamics. Proc. Natl. Acad. Sci. U. S. A. 2015, 112, 13567–13572. 10.1073/pnas.1508584112. 26487681 PMC4640757

[ref26] Morcos F.; Jana B.; Hwa T.; Onuchic J. N. Coevolutionary Signals across Protein Lineages Help Capture Multiple Protein Conformations. Proc. Natl. Acad. Sci. U. S. A. 2013, 110, 20533–20538. 10.1073/pnas.1315625110. 24297889 PMC3870752

[ref27] Tian P.; Boomsma W.; Wang Y.; Otzen D. E.; Jensen M. H.; Lindorff-Larsen K. Structure of a Functional Amyloid Protein Subunit Computed Using Sequence Variation. J. Am. Chem. Soc. 2015, 22 10.1021/ja5093634. 25415595

[ref28] Kassem M. M.; Wang Y.; Boomsma W.; Lindorff-Larsen K. Structure of the Bacterial Cytoskeleton Protein Bactofilin by NMR Chemical Shifts and Sequence Variation. Biophys. J. 2016, 110, 2342–2348. 10.1016/j.bpj.2016.04.039. 27276252 PMC4922582

[ref29] Lui S.; Tiana G. The Network of Stabilizing Contacts in Proteins Studied by Coevolutionary Data. J. Chem. Phys. 2013, 139, 155103 10.1063/1.4826096. 24160546

[ref30] Contini A.; Tiana G. A Many-Body Term Improves the Accuracy of Effective Potentials Based on Protein Coevolutionary Data. J. Chem. Phys. 2015, 143, 025103 10.1063/1.4926665. 26178131

[ref31] Liwo A.; Khalili M.; Scheraga H. A. Ab Initio Simulations of Protein-Folding Pathways by Molecular Dynamics with the United-Residue Model of Polypeptide Chains. Proc. Natl. Acad. Sci. U. S. A. 2005, 102, 2362–2367. 10.1073/pnas.0408885102. 15677316 PMC548970

[ref32] Kmiecik S.; Kolinski A. Characterization of Protein-Folding Pathways by Reduced-Space Modeling. Proc. Natl. Acad. Sci. 2007, 104, 12330–12335. 10.1073/pnas.0702265104. 17636132 PMC1941469

[ref33] Voegler Smith A.; Hall C. K. α-Helix Formation: Discontinuous Molecular Dynamics on an Intermediate-Resolution Protein Model. Proteins: Struct., Funct., Bioinf. 2001, 44, 344–360. 10.1002/prot.1100. 11455608

[ref34] Ekeberg M.; Lövkvist C.; Lan Y.; Weigt M.; Aurell E. Improved Contact Prediction in Proteins: Using Pseudolikelihoods to Infer Potts Models. Phys. Rev. E 2013, 87, 012707 10.1103/PhysRevE.87.012707. 23410359

[ref35] Fantini M.; Malinverni D.; De Los Rios P.; Pastore A. New Techniques for Ancient Proteins: Direct Coupling Analysis Applied on Proteins Involved in Iron Sulfur Cluster Biogenesis. Front. Mol. Biosci. 2017, 4, 40 10.3389/fmolb.2017.00040. 28664160 PMC5471300

[ref36] Ekeberg M.; Hartonen T.; Aurell E. Fast Pseudolikelihood Maximization for Direct-Coupling Analysis of Protein Structure from Many Homologous Amino-Acid Sequences. J. Comput. Phys. 2014, 276, 341–356. 10.1016/j.jcp.2014.07.024.

[ref37] Miyazawa S.; Jernigan R. L. Estimation of Effective Interresidue Contact Energies from Protein Crystal Structures: Quasi-Chemical Approximation. Macromolecules 1985, 18, 534–552. 10.1021/ma00145a039.

[ref38] Franco G.; Cagiada M.; Bussi G.; Tiana G. Statistical Mechanical Properties of Sequence Space Determine the Efficiency of the Various Algorithms to Predict Interaction Energies and Native Contacts from Protein Coevolution. Phys. Biol. 2019, 16, 046007 10.1088/1478-3975/ab1c15. 31018179

[ref39] McGuffin L. J.; Bryson K.; Jones D. T. The PSIPRED Protein Structure Prediction Server. Bioinformatics 2000, 16, 404–405. 10.1093/bioinformatics/16.4.404. 10869041

[ref40] Tiana G.; Villa F.; Zhan Y.; Capelli R.; Paissoni C.; Sormanni P.; Heard E.; Giorgetti L.; Meloni R. MonteGrappa: An Iterative Monte Carlo Program to Optimize Biomolecular Potentials in Simplified Models. Comput. Phys. Commun. 2015, 186, 93–104. 10.1016/j.cpc.2014.09.012.

[ref41] Swendsen R. H.; Wang J. S. Replica Monte Carlo Simulation of Spin Glasses. Phys. Rev. Lett. 1986, 57, 2607–2609. 10.1103/PhysRevLett.57.2607. 10033814

[ref42] Torrie G. M.; Valleau J. P. Nonphysical Sampling Distributions in Monte Carlo Free-Energy Estimation: Umbrella Sampling. J. Comput. Phys. 1977, 23, 187–199. 10.1016/0021-9991(77)90121-8.

[ref43] Oliveira L. C.; Schug A.; Onuchic J. N. Geometrical Features of the Protein Folding Mechanism Are a Robust Property of the Energy Landscape: A Detailed Investigation of Several Reduced Models. J. Phys. Chem. B 2008, 6131 10.1021/jp0769835. 18251535

[ref44] Chan H. S. Modeling Protein Density of States: Additive Hydrophobic Effects Are Insufficient for Calorimetric Two-State Cooperativity. Proteins: Struct., Funct., Genet. 2000, 40, 543–571. 10.1002/1097-0134(20000901)40:4<543::AID-PROT20>3.0.CO;2-O. 10899781

[ref45] Davila S.; Liu P.; Smith A.; Marshall A. G.; Pedigo S. Spontaneous Calcium-Independent Dimerization of the Isolated First Domain of Neural Cadherin. Biochemistry 2018, 57, 6404–6415. 10.1021/acs.biochem.8b00733. 30387993

[ref46] Parisini E.; Higgins J. M. G.; Liu J.; Brenner M. B.; Wang J. The Crystal Structure of Human E-Cadherin Domains 1 and 2, and Comparison with Other Cadherins in the Context of Adhesion Mechanism. J. Mol. Biol. 2007, 373, 401–411. 10.1016/j.jmb.2007.08.011. 17850815 PMC2094043

[ref47] Kudo S.; Caaveiro J. M. M.; Tsumoto K. Adhesive Dimerization of Human P-Cadherin Catalyzed by a Chaperone-like Mechanism. Structure 2016, 24, 1523 10.1016/j.str.2016.07.002. 27545624

[ref48] Li Y.; Altorelli N. L.; Bahna F.; Honig B.; Shapiro L.; Palmer A. G. Mechanism of E-Cadherin Dimerization Probed by NMR Relaxation Dispersion. Proc. Natl. Acad. Sci. U. S. A. 2013, 110, 16462–16467. 10.1073/pnas.1314303110. 24067646 PMC3799306

[ref49] Kudo S.; Caaveiro J. M. M.; Goda S.; Nagatoishi S.; Ishii K.; Matsuura T.; Sudou Y.; Kodama T.; Hamakubo T.; Tsumoto K. Identification and Characterization of the X-Dimer of Human P-Cadherin: Implications for Homophilic Cell Adhesion. Biochemistry 2014, 53, 1742–1752. 10.1021/bi401341g. 24559158

[ref50] Tariq H.; Bella J.; Jowitt T. A.; Holmes D. F.; Rouhi M.; Nie Z.; Baldock C.; Garrod D.; Tabernero L. Cadherin Flexibility Provides a Key Difference between Desmosomes and Adherens Junctions. Proc. Natl. Acad. Sci. U. S. A. 2015, 112, 5395–5400. 10.1073/pnas.1420508112. 25855637 PMC4418904

[ref51] Bajpai S.; Correia J.; Feng Y.; Figueiredo J.; Sun S. X.; Longmore G. D.; Suriano G.; Wirtz D. α-Catenin Mediates Initial E-Cadherin-Dependent Cell-Cell Recognition and Subsequent Bond Strengthening. Proc. Natl. Acad. Sci. U. S. A. 2008, 105, 18331–18336. 10.1073/pnas.0806783105. 19017792 PMC2587611

[ref52] Pitera J. W.; Chodera J. D. On the Use of Experimental Observations to Bias Simulated Ensembles. J. Chem. Theory Comput. 2012, 8, 3445–3451. 10.1021/ct300112v. 26592995

[ref53] Tiana G.; Giorgetti L. Integrating Experiment, Theory and Simulation to Determine the Structure and Dynamics of Mammalian Chromosomes. Curr. Opin. Struct. Biol. 2018, 49, 11–17. 10.1016/j.sbi.2017.10.016. 29128709

[ref54] Gueudré T.; Baldassi C.; Zamparo M.; Weigt M.; Pagnani A. Simultaneous Identification of Specifically Interacting Paralogs and Interprotein Contacts by Direct Coupling Analysis. Proc. Natl. Acad. Sci. U. S. A. 2016, 113, 12186–12191. 10.1073/pnas.1607570113. 27729520 PMC5087065

[ref55] Noel J. K.; Whitford P. C.; Sanbonmatsu K. Y.; Onuchic J. N. SMOG@ctbp: Simplified Deployment of Structure-Based Models in GROMACS. Nucleic Acids Res. 2010, 38, W657–W661. 10.1093/nar/gkq498. 20525782 PMC2896113

[ref56] Boomsma W.; Ferkinghoff-Borg J.; Lindorff-Larsen K. Combining Experiments and Simulations Using the Maximum Entropy Principle. PLoS Comput. Biol. 2014, 10, e1003406 10.1371/journal.pcbi.1003406. 24586124 PMC3930489

