# Supplementary material for: Molecular Recognition between Cadherins Studied by a Coarse-Grained Model Interacting with a Coevolutionary Potential
Source: J Phys Chem B. 2020 Apr 27;124(20):4079–88. doi: 10.1021/acs.jpcb.0c01671 (PMC8007105; doi:10.1021/acs.jpcb.0c01671)
Supplement: Supplementary file 1 — jp0c01671_si_001.pdf [file jp0c01671_si_001.pdf]

Supporting Information

Molecular Recognition between Cadherins Studied by Coevolutionary Potential

Sara Terzoli and Guido Tiana
Department of Physics and Center for Complexity and Biosystems, Università degli Studi di Milano and INFN, via Celoria 16, 20133 Milano, Italy

S1 The pseudolikelihood approximation

Given an alignment $\{\sigma_i^b\}$ of B homologous sequences and the empirical frequencies $f_i(\sigma)$ and $f_{ij}(\sigma,\pi)$ obtained from $\{\sigma_i^b\}$, the parameters $h_i(\sigma)$ and $J_{ij}(\sigma,\pi)$ of a Potts-like model of interaction

$$U(\{\sigma_i^b\}) = \sum_i h_i(\sigma_i) + \sum_{i<j} J_{ij}(\sigma_i,\sigma_j) \quad \text{(S1)}$$

are obtained maximizing the log-pseudolikelihood

$$l_{ps} = \frac{1}{B}\sum_b log\left(\sum_\pi exp\left[h_i(\pi) + \sum_{j\neq i} J_{ij}(\pi,\sigma_j)\right]\right) - \sum_i\sum_\pi f_i(\pi)h_i(\pi) - \sum_{i\neq j}\sum_{\pi\tau} f_{ij}(\pi,\tau)J_{ij}(\pi,\tau) \quad \text{(S2)}$$

as discussed in ref. 1. The maximization is constrained by two regularizers

$$\lambda\sum_{i\sigma} h_i(\sigma)^2 + \alpha\sum_{ij\sigma\pi}\left(J_{ij}(\sigma,\pi) - \varepsilon(\sigma,\tau)\right)^2, \quad \text{(S3)}$$

where $\varepsilon(\sigma,\tau)$ is some *a priori* energies we have for the system. Calling $\delta J_{ij}(\sigma,\pi) = J_{ij}(\sigma,\pi) - \varepsilon(\sigma,\tau)$ and substituting in Eq. (S2) one can realize that this procedure is equivalent to finding the energy corrections $\delta J_{ij}(\sigma,\pi)$ to the $\varepsilon(\sigma,\tau)$ under the standard l_2 regularizer centered around zero.

S2 Tuning of the energy metaparameters

To find realistic values for α, ε_0 and ε_{dih} , we have explored their parameter space using bovine pancreatic trypsin inhibitor (BPTI, pdb code 1BPI) instead of cadherins themselves, because of the much smaller size of the former.
First, we required that the monomeric protein stays in the crystallographic native conformation at low temperature. Figure S3 displays the average RMSD with respect to the native conformation of BPTI as a function of temperature, calculated for different choices of the Lagrange multiplier α that controls the l_2 normalizer and of the parameter ε_0 that sets the zero of the *a priori* statistical potential (cf. Eq. 3). The Lagrange multiplier λ on the fields (cf. Eq. S3) is kept fixed at the value 0.1, defined optimal in ref. 2, and the potential on the dihedrals is switched off. For $\alpha=10^{-5}$ and $\varepsilon_0=-1$, the average RMSD stays at the lowest value of ≈0.25 nm in a large temperature interval; thus, we used these values in the rest of the work.
Then, we performed the same kind of simulations applying the potential on the dihedrals of the backbone and varying ε_{dih}. The results are reported in Fig. S4. The lowest average RMSD is obtained for $\varepsilon_{dih}=90$, and consequently we used this value for the rest of the calculations. The energy associated with each dihedral term is then quite large (the energy scale associated with two-body interactions is 1) To investigate the possibility that at $\varepsilon_{dih}=90$ the properties of the chain are only controlled by the dihedral term, we performed a control simulation (gray symbols in Fig. S4) in which the parameters of the two-body interaction are randomly reshuffled, while the dihedral terms are the correct ones. As expected, the average RMSD of the protein increases drastically.

Finally, we performed a parallel-tempering simulation on BPTI and on another protein, acyl-coenzime A binding protein (ACBP, pdb code 2ABD) on a wider range of temperature to study the folding transition of test proteins within the model. In this range of temperatures, both proteins display a marked increase in the average RMSD (cf. Fig. S5a). The specific heat of BPTI, calculated from the multiple-histogram method[3], displays a low-temperature peak around T=5, corresponding to freezing of the chain in the lowest-energy state (cf. Fig. S5b), a denaturation peak around T=10 and a small peak associated to swelling at larger temperature. ACBP displays the same freezing peak and a much broader peak associated with denaturation and swelling.
Again, to study the role of the different terms in the potential, we plotted in Fig. S5c the average two-body energy and the average dihedral energy. Upon increasing the temperature, the two-body term follows closely the total energy, while the dihedral term displays milder changes. Moreover, the two terms seem poorly correlated in the visited conformations (cf. Fig. S5d). Even if the number of dihedral terms in the potential is much smaller (~N) than that of the two-body terms (~N^2), the large contribution of each of them suggests that the dihedral term gives a non-trivial, but important (cf. Fig. S4), contribution to the stabilization of the proteins.

S3 References

1. Ekeberg M, Lövkvist C, Lan Y, Weigt M, Aurell E. Improved contact prediction in proteins: Using pseudolikelihoods to infer Potts models. Phys. Rev. E, Stat. nonlinear, soft matter Phys. 2013;87:620630.
2. Fantini M, Malinverni D, De Los Rios P, Pastore A. New Techniques for Ancient Proteins: Direct Coupling Analysis Applied on Proteins Involved in Iron Sulfur Cluster Biogenesis. Front. Mol. Biosci. 2017;4:40.
3. Ferrenberg A, Swendsen R. Optimized Monte Carlo data analysis. Phys. Rev. Lett. 1989;63:1195–1198.
4. Vendome J, Felsovalyi K, Song H, Yang Z, Jin X, Brasch J, Harrison OJ, Ahlsén G, Bahna F, Kaczynska A, et al. Structural and energetic determinants of adhesive binding specificity in type I cadherins. Proc. Natl. Acad. Sci. U. S. A. 2014;111:E4175–E4184.

System	K_D simulation	K_D experiment[4]
NN	2.0	26
PP	35.1	31
EE	81.5	96
PE	14.0	~50
NE	>100	~50
PN	>100	>100

Table S1: comparison between the dissociation constants (in μM) obtained from the simulation and those obtained by analytical ultracentrifugation or plasmon resonance experiments for the EC1-2 system of different cadherins. The K_D is calculated from the simulations counting the fraction of frames in which the two chains contacts (considering a lower threshold on inter-chain contact energies of -26 to define bound conformations) and knowing that the side of the box is 20 nm.

Resid	α	β
1	0.999	0.000
2	0.824	0.017
3	0.800	0.009
4	0.925	0.008
5	0.970	0.006
6	0.936	0.011
7	0.233	0.003
8	0.126	0.006
9	0.727	0.003
10	0.651	0.007
11	0.916	0.020
12	0.957	0.029
13	0.976	0.012
14	0.978	0.008
15	0.981	0.006
16	0.960	0.008
17	0.880	0.009
18	0.863	0.004
19	0.099	0.002
20	0.028	0.001
21	0.021	0.000
22	0.020	0.000
23	0.019	0.000
24	0.032	0.001
25	0.115	0.004
26	0.809	0.006
27	0.951	0.021
28	0.950	0.024
29	0.965	0.015
30	0.927	0.052
31	0.884	0.025
32	0.467	0.006
33	0.078	0.003
34	0.019	0.000
35	0.018	0.000
36	0.019	0.000
37	0.018	0.000
38	0.024	0.001
39	0.103	0.004
40	0.960	0.005
41	0.987	0.010
42	0.984	0.009
43	0.974	0.010
44	0.973	0.009
45	0.965	0.009
46	0.978	0.006
47	0.968	0.010
48	0.837	0.023
49	0.692	0.014
50	0.817	0.004
51	0.099	0.002
52	0.015	0.001
53	0.024	0.001
54	0.465	0.003
55	0.974	0.010
56	0.990	0.010
57	0.955	0.021
58	0.790	0.004
59	0.068	0.002
60	0.012	0.000
61	0.014	0.001
62	0.061	0.002
63	0.965	0.004
64	0.979	0.012
65	0.962	0.014
66	0.900	0.022
67	0.828	0.026
68	0.790	0.025
69	0.775	0.024
70	0.721	0.030
71	0.851	0.012
72	0.800	0.004
73	0.057	0.001
74	0.015	0.000
75	0.017	0.000
76	0.021	0.000
77	0.023	0.000
78	0.024	0.000
79	0.022	0.000
80	0.021	0.000
81	0.028	0.001
82	0.264	0.005
83	0.986	0.006
84	0.993	0.008
85	0.974	0.014
86	0.969	0.007
87	0.855	0.012
88	0.703	0.009
89	0.426	0.005
90	0.246	0.004
91	0.859	0.002
92	0.070	0.001
93	0.013	0.000
94	0.018	0.000
95	0.024	0.000
96	0.024	0.000
97	0.026	0.000
98	0.027	0.001
99	0.158	0.004
100	0.887	0.005
101	0.946	0.030
102	0.975	0.017
103	0.974	0.011
104	0.962	0.012
105	0.972	0.009
106	0.966	0.006
107	0.556	0.003
108	0.520	0.006
109	0.705	0.016
110	0.813	0.035
111	0.785	0.022
112	0.281	0.006
113	0.077	0.002
114	0.031	0.001
115	0.019	0.000
116	0.026	0.001
117	0.152	0.006
118	0.626	0.006
119	0.955	0.017
120	0.986	0.012
121	0.979	0.009
122	0.975	0.007
123	0.982	0.005
124	0.983	0.005
125	0.875	0.005
126	0.068	0.001
127	0.022	0.000
128	0.017	0.000
129	0.020	0.000
130	0.025	0.000
131	0.020	0.000
132	0.015	0.000
133	0.030	0.001
134	0.480	0.003
135	0.978	0.010
136	0.984	0.011
137	0.976	0.012
138	0.967	0.014
139	0.891	0.072
140	0.779	0.151
141	0.726	0.059
142	0.790	0.009
143	0.137	0.003
144	0.030	0.001
145	0.017	0.000
146	0.020	0.000
147	0.019	0.000
148	0.020	0.000
149	0.039	0.001
150	0.300	0.006
151	0.797	0.009
152	0.973	0.013
153	0.983	0.009
154	0.980	0.008
155	0.978	0.008
156	0.977	0.008
157	0.974	0.009
158	0.959	0.013
159	0.939	0.032
160	0.944	0.018
161	0.869	0.014
162	0.188	0.003
163	0.028	0.001
164	0.029	0.002
165	0.281	0.006
166	0.981	0.007
167	0.990	0.011
168	0.983	0.010
169	0.979	0.007
170	0.902	0.003
171	0.051	0.001
172	0.015	0.001
173	0.026	0.001
174	0.201	0.006
175	0.954	0.007
176	0.949	0.023
177	0.954	0.016
178	0.894	0.014
179	0.912	0.016
180	0.684	0.030
181	0.475	0.027
182	0.567	0.015
183	0.906	0.010
184	0.684	0.005
185	0.074	0.002
186	0.017	0.001
187	0.017	0.000
188	0.022	0.000
189	0.023	0.000
190	0.024	0.000
191	0.022	0.000
192	0.020	0.000
193	0.034	0.001
194	0.392	0.004
195	0.984	0.007
196	0.991	0.009
197	0.973	0.012
198	0.973	0.008
199	0.955	0.017
200	0.902	0.040
201	0.848	0.054
202	0.847	0.024
203	0.715	0.009
204	0.677	0.005
205	0.280	0.005
206	0.065	0.002
207	0.064	0.001
208	0.041	0.001
209	0.022	0.000
210	0.025	0.000
211	0.023	0.000
212	0.024	0.000
213	0.041	0.001
214	0.210	0.002
215	0.999	0.000

Table S2: secondary structure propensities for N-cadherin

Resid	α	β
1	0.999	0.001
2	0.829	0.018
3	0.791	0.012
4	0.944	0.008
5	0.980	0.005
6	0.964	0.007
7	0.167	0.002
8	0.060	0.003
9	0.245	0.006
10	0.238	0.009
11	0.848	0.031
12	0.943	0.043
13	0.958	0.026
14	0.970	0.011
15	0.976	0.008
16	0.967	0.007
17	0.917	0.010
18	0.924	0.005
19	0.276	0.003
20	0.076	0.003
21	0.026	0.001
22	0.016	0.000
23	0.019	0.000
24	0.058	0.002
25	0.155	0.007
26	0.820	0.005
27	0.884	0.019
28	0.911	0.024
29	0.935	0.035
30	0.876	0.104
31	0.882	0.051
32	0.667	0.008
33	0.304	0.004
34	0.053	0.002
35	0.018	0.000
36	0.017	0.000
37	0.017	0.000
38	0.020	0.001
39	0.072	0.003
40	0.882	0.004
41	0.977	0.014
42	0.982	0.011
43	0.973	0.011
44	0.973	0.009
45	0.962	0.010
46	0.976	0.006
47	0.957	0.010
48	0.853	0.015
49	0.530	0.009
50	0.617	0.003
51	0.105	0.003
52	0.015	0.001
53	0.023	0.001
54	0.306	0.004
55	0.981	0.007
56	0.990	0.011
57	0.959	0.020
58	0.841	0.005
59	0.062	0.002
60	0.012	0.000
61	0.015	0.001
62	0.056	0.002
63	0.939	0.004
64	0.981	0.012
65	0.953	0.017
66	0.895	0.022
67	0.876	0.019
68	0.752	0.023
69	0.608	0.014
70	0.551	0.006
71	0.891	0.007
72	0.798	0.005
73	0.051	0.001
74	0.015	0.000
75	0.017	0.000
76	0.022	0.000
77	0.024	0.000
78	0.024	0.000
79	0.022	0.000
80	0.021	0.000
81	0.029	0.001
82	0.280	0.004
83	0.987	0.006
84	0.993	0.009
85	0.961	0.016
86	0.966	0.007
87	0.856	0.012
88	0.619	0.007
89	0.209	0.006
90	0.178	0.004
91	0.873	0.002
92	0.079	0.001
93	0.015	0.000
94	0.017	0.000
95	0.024	0.000
96	0.025	0.000
97	0.025	0.000
98	0.027	0.001
99	0.084	0.003
100	0.870	0.003
101	0.897	0.019
102	0.960	0.019
103	0.977	0.012
104	0.968	0.012
105	0.972	0.009
106	0.966	0.007
107	0.821	0.010
108	0.772	0.042
109	0.722	0.062
110	0.717	0.037
111	0.781	0.020
112	0.490	0.006
113	0.184	0.005
114	0.046	0.002
115	0.018	0.000
116	0.019	0.000
117	0.038	0.002
118	0.174	0.009
119	0.923	0.014
120	0.980	0.014
121	0.982	0.010
122	0.974	0.008
123	0.984	0.004
124	0.984	0.005
125	0.878	0.005
126	0.107	0.002
127	0.048	0.001
128	0.021	0.000
129	0.017	0.000
130	0.025	0.000
131	0.021	0.000
132	0.015	0.000
133	0.030	0.001
134	0.492	0.003
135	0.967	0.014
136	0.985	0.016
137	0.969	0.021
138	0.947	0.030
139	0.865	0.137
140	0.832	0.142
141	0.723	0.144
142	0.441	0.127
143	0.431	0.053
144	0.149	0.030
145	0.043	0.003
146	0.039	0.003
147	0.027	0.001
148	0.019	0.001
149	0.020	0.001
150	0.121	0.006
151	0.612	0.014
152	0.801	0.036
153	0.953	0.023
154	0.977	0.009
155	0.969	0.016
156	0.972	0.012
157	0.968	0.014
158	0.950	0.029
159	0.870	0.109
160	0.814	0.171
161	0.902	0.050
162	0.870	0.015
163	0.127	0.002
164	0.020	0.001
165	0.025	0.002
166	0.341	0.004
167	0.978	0.008
168	0.989	0.012
169	0.975	0.019
170	0.971	0.004
171	0.152	0.002
172	0.018	0.001
173	0.016	0.001
174	0.051	0.001
175	0.821	0.004
176	0.917	0.023
177	0.950	0.028
178	0.938	0.017
179	0.838	0.015
180	0.847	0.029
181	0.646	0.023
182	0.451	0.011
183	0.535	0.008
184	0.950	0.010
185	0.965	0.005
186	0.059	0.001
187	0.016	0.001
188	0.016	0.000
189	0.023	0.000
190	0.023	0.000
191	0.023	0.000
192	0.020	0.000
193	0.020	0.000
194	0.041	0.002
195	0.553	0.003
196	0.967	0.013
197	0.987	0.011
198	0.973	0.014
199	0.963	0.010
200	0.948	0.009
201	0.849	0.011
202	0.730	0.006
203	0.273	0.003
204	0.027	0.001
205	0.017	0.000
206	0.022	0.000
207	0.024	0.000
208	0.027	0.000
209	0.023	0.000
210	0.023	0.000
211	0.037	0.001
212	0.157	0.003
213	1.000	0.000

Table S3: secondary structure propensities for E-cadherin

Resid	α	β
1	0.999	0.001
2	0.842	0.017
3	0.526	0.009
4	0.433	0.007
5	0.720	0.005
6	0.819	0.011
7	0.729	0.014
8	0.401	0.007
9	0.142	0.007
10	0.848	0.002
11	0.721	0.006
12	0.900	0.014
13	0.953	0.019
14	0.977	0.010
15	0.978	0.008
16	0.980	0.006
17	0.961	0.007
18	0.847	0.009
19	0.834	0.004
20	0.077	0.001
21	0.021	0.001
22	0.026	0.001
23	0.022	0.000
24	0.021	0.000
25	0.058	0.002
26	0.213	0.006
27	0.751	0.008
28	0.944	0.019
29	0.967	0.016
30	0.963	0.016
31	0.903	0.056
32	0.927	0.021
33	0.649	0.007
34	0.180	0.004
35	0.030	0.001
36	0.017	0.000
37	0.018	0.000
38	0.020	0.000
39	0.045	0.001
40	0.128	0.007
41	0.957	0.005
42	0.988	0.010
43	0.984	0.009
44	0.966	0.013
45	0.974	0.009
46	0.974	0.008
47	0.979	0.007
48	0.943	0.030
49	0.791	0.028
50	0.708	0.015
51	0.886	0.003
52	0.100	0.002
53	0.015	0.001
54	0.034	0.002
55	0.718	0.003
56	0.972	0.012
57	0.983	0.016
58	0.931	0.023
59	0.774	0.003
60	0.057	0.001
61	0.012	0.001
62	0.014	0.001
63	0.066	0.002
64	0.968	0.004
65	0.981	0.013
66	0.972	0.012
67	0.920	0.017
68	0.920	0.012
69	0.790	0.018
70	0.672	0.026
71	0.674	0.041
72	0.812	0.018
73	0.750	0.005
74	0.087	0.002
75	0.018	0.000
76	0.016	0.000
77	0.021	0.000
78	0.023	0.000
79	0.026	0.000
80	0.022	0.000
81	0.020	0.000
82	0.034	0.001
83	0.401	0.003
84	0.984	0.008
85	0.992	0.009
86	0.961	0.016
87	0.970	0.006
88	0.905	0.013
89	0.795	0.010
90	0.283	0.006
91	0.228	0.004
92	0.895	0.002
93	0.166	0.002
94	0.017	0.000
95	0.016	0.000
96	0.023	0.000
97	0.025	0.000
98	0.024	0.000
99	0.022	0.001
100	0.073	0.002
101	0.786	0.003
102	0.906	0.019
103	0.969	0.016
104	0.979	0.010
105	0.972	0.009
106	0.974	0.008
107	0.973	0.005
108	0.566	0.006
109	0.474	0.004
110	0.444	0.004
111	0.235	0.005
112	0.670	0.008
113	0.587	0.013
114	0.326	0.008
115	0.198	0.006
116	0.084	0.004
117	0.070	0.003
118	0.117	0.009
119	0.414	0.015
120	0.907	0.048
121	0.980	0.017
122	0.979	0.010
123	0.975	0.007
124	0.983	0.005
125	0.982	0.005
126	0.827	0.005
127	0.086	0.001
128	0.020	0.001
129	0.016	0.000
130	0.021	0.000
131	0.024	0.000
132	0.020	0.000
133	0.016	0.000
134	0.028	0.001
135	0.283	0.004
136	0.979	0.009
137	0.988	0.014
138	0.974	0.016
139	0.862	0.118
140	0.771	0.207
141	0.722	0.212
142	0.707	0.077
143	0.626	0.029
144	0.775	0.009
145	0.363	0.006
146	0.068	0.003
147	0.032	0.001
148	0.020	0.000
149	0.018	0.000
150	0.026	0.001
151	0.214	0.006
152	0.737	0.006
153	0.925	0.019
154	0.979	0.012
155	0.981	0.008
156	0.978	0.009
157	0.972	0.010
158	0.967	0.011
159	0.915	0.041
160	0.791	0.183
161	0.801	0.170
162	0.904	0.046
163	0.901	0.015
164	0.254	0.004
165	0.037	0.002
166	0.031	0.002
167	0.187	0.005
168	0.978	0.007
169	0.988	0.010
170	0.961	0.028
171	0.958	0.003
172	0.084	0.001
173	0.015	0.000
174	0.017	0.000
175	0.043	0.001
176	0.514	0.004
177	0.900	0.016
178	0.960	0.040
179	0.947	0.021
180	0.896	0.017
181	0.925	0.013
182	0.768	0.021
183	0.615	0.017
184	0.717	0.009
185	0.951	0.008
186	0.914	0.006
187	0.101	0.002
188	0.022	0.001
189	0.016	0.000
190	0.022	0.000
191	0.023	0.000
192	0.024	0.000
193	0.021	0.000
194	0.021	0.000
195	0.032	0.001
196	0.264	0.004
197	0.982	0.006
198	0.991	0.009
199	0.969	0.016
200	0.965	0.009
201	0.947	0.008
202	0.767	0.010
203	0.627	0.005
204	0.095	0.003
205	0.022	0.000
206	0.020	0.000
207	0.022	0.000
208	0.024	0.000
209	0.027	0.000
210	0.024	0.000
211	0.025	0.000
212	0.039	0.001
213	0.174	0.002
214	0.999	0.000

Table S4: secondary structure propensities for P-cadherin

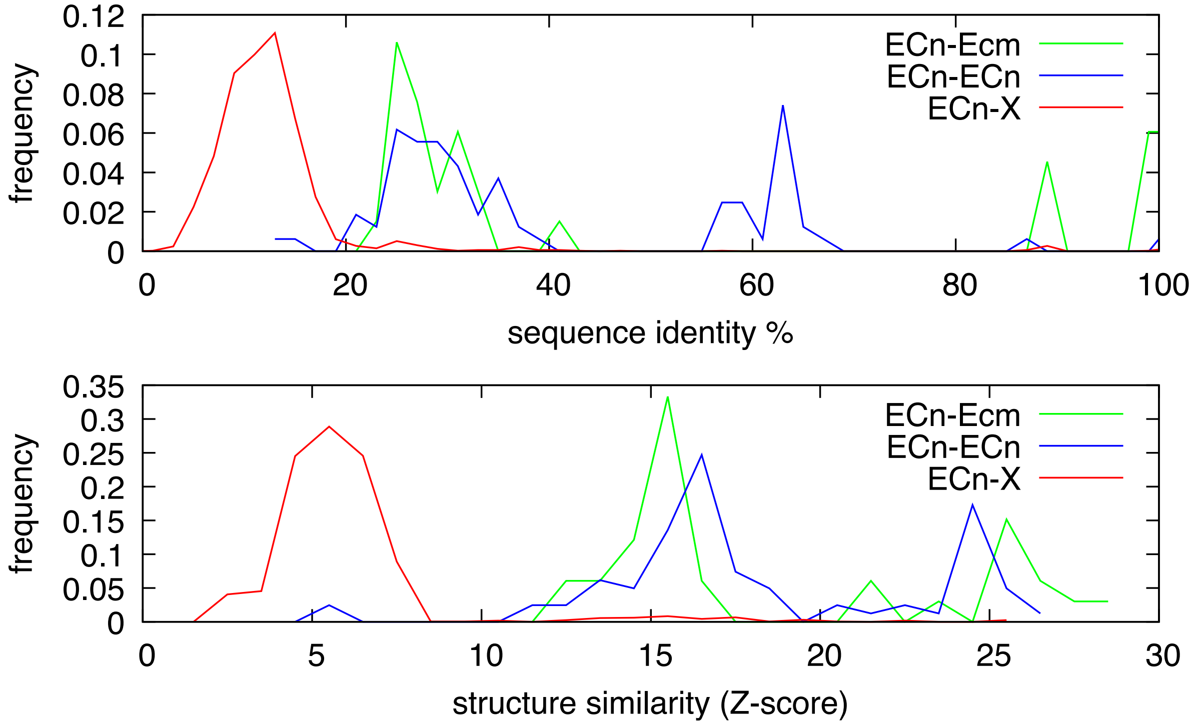

Figure S1: Sequence (above panel) and structural (below panel) similarity between the same domain of different cadherins, between different domains of the same cadherin type and between cadherin domains and, as a control (X), between cadherin domains and structurally similar domains (Z>2), as calculated from the Dali database (http://ekhidna2.biocenter.helsinki.fi/dali/).

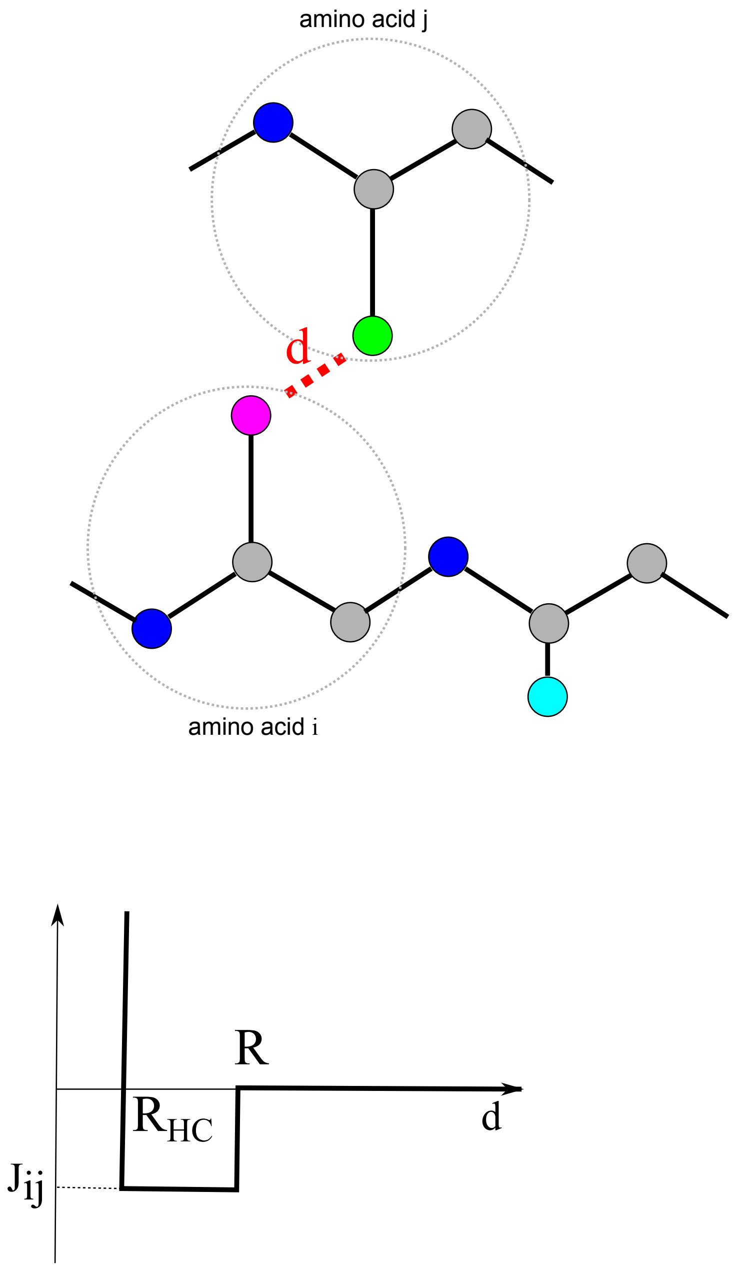

Figure S2: a sketch of the model. Each amino acid is described by four beads, representing the atoms N, CA, C and the side chain, respectively. The interaction between two amino acids depends on the distance *d* between the side chains, and has the form of a spherical well with range R, hard-core radius R_{HC} and depth J_{ij} depending on the specific pair. Each pair of atoms have a hard-core repulsion.

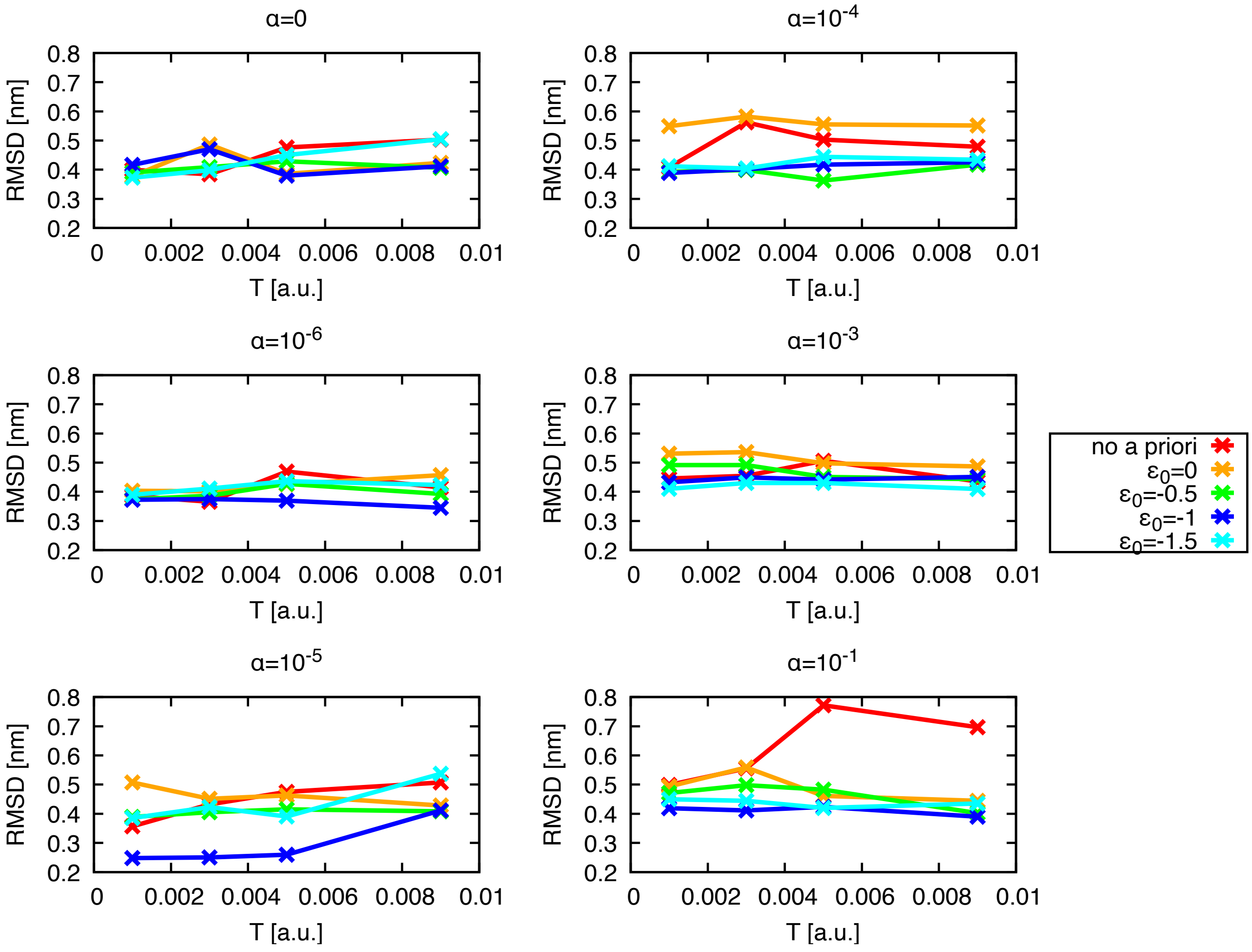

Figure S3: the average RMSD of BPTI as a function of temperature T, obtained at various values of the Lagrange multiplier α that controls the l_2 normalizer and of the parameter ε_0 that sets the zero of the *a priori* statistical potential. In the case with "no a priori" we used a l_2 normalizer centered at zero.

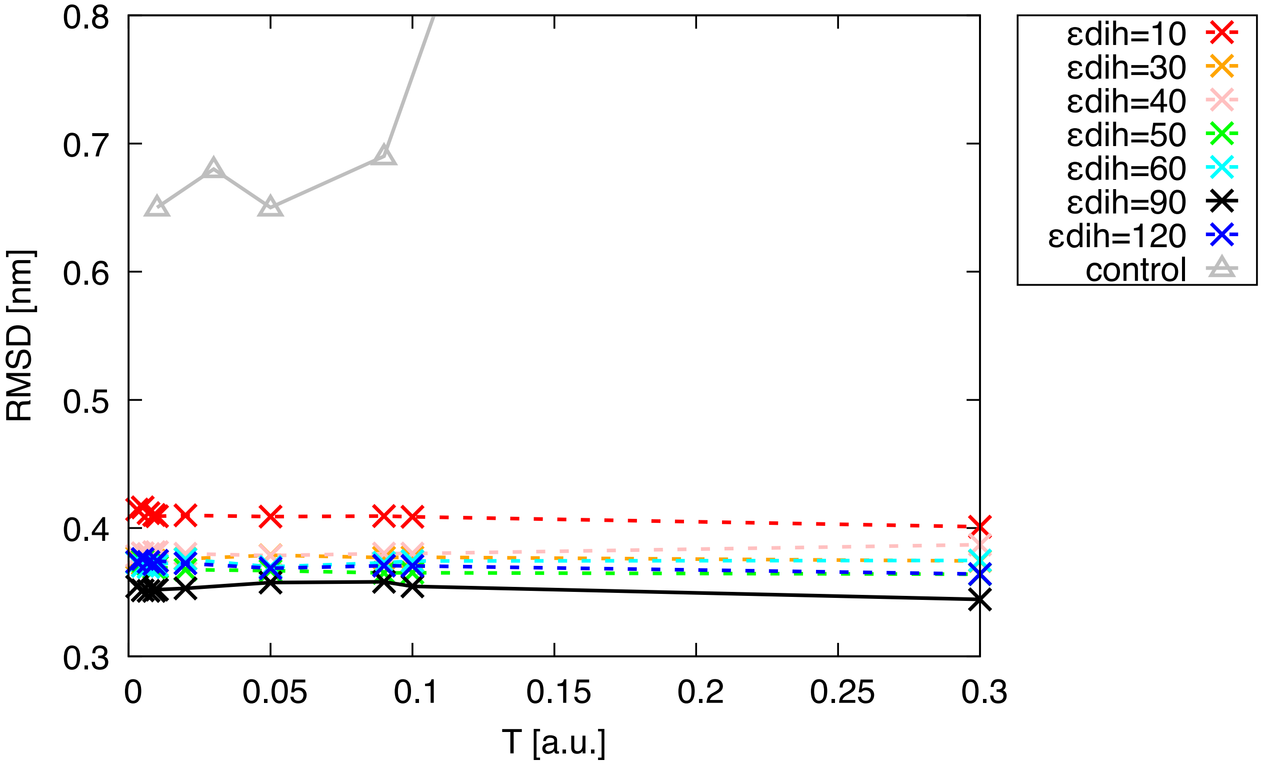

Figure S4: the average RMSD of BPTI as a function of temperature T using $\alpha=10^{-5}$ and $\varepsilon_0=-1$, for different choices of ε_{dih}, that controls the potential on the dihedrals. The control points are obtained reshuffling at random the parameters of the two-body potentials, and keeping the correct dihedral potential with $\varepsilon_{dih}=90$.

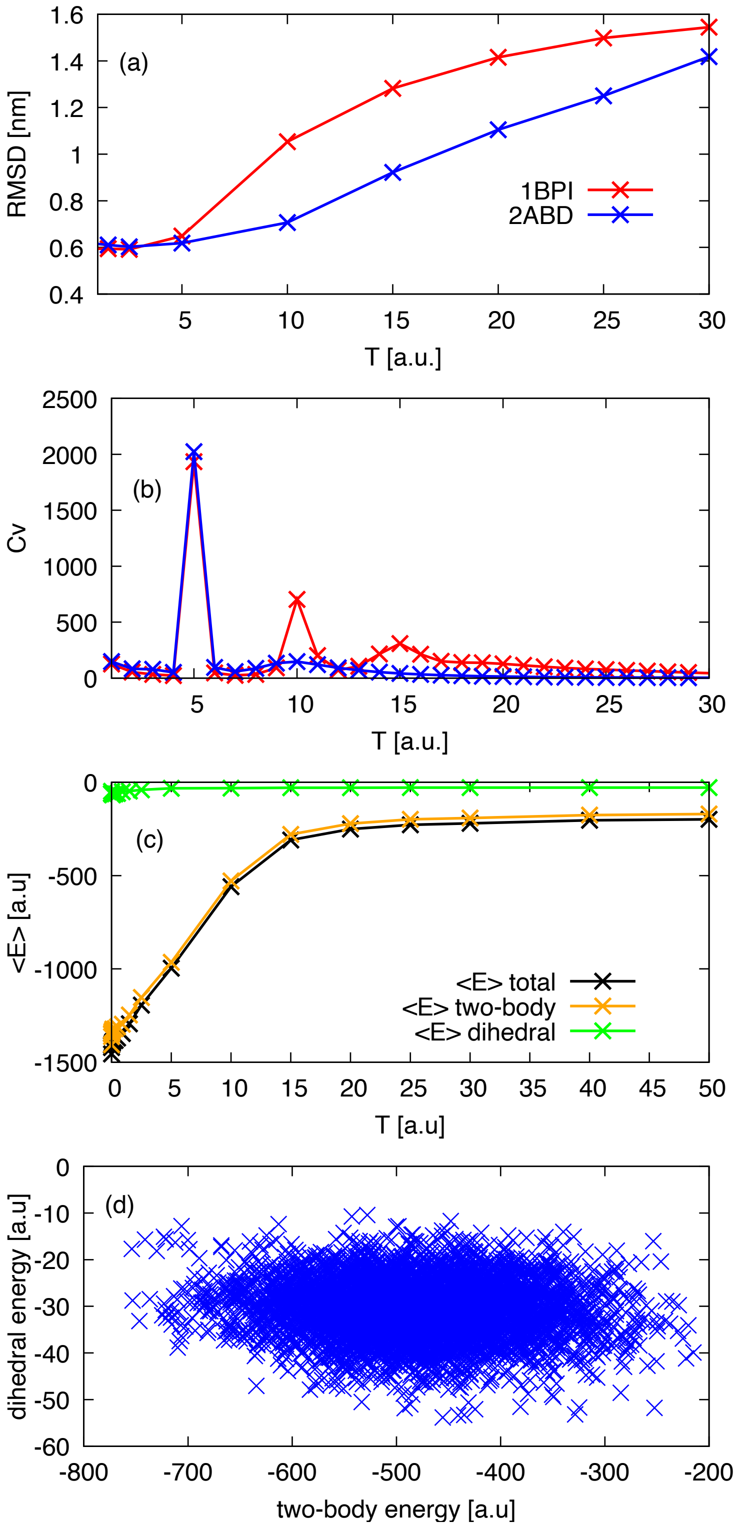

Figure S5: The average RMSD (a) and the heat capacity C_V (b) of BPTI and ACBP as a function of temperature, calculated from parallel-tempering simulations. (c) The two-body and the dihedral terms of the total energy for BPTI at various temperatures. (d) A scatter plot of the two-body and of the dihedral terms of the energy for conformations at T=5.

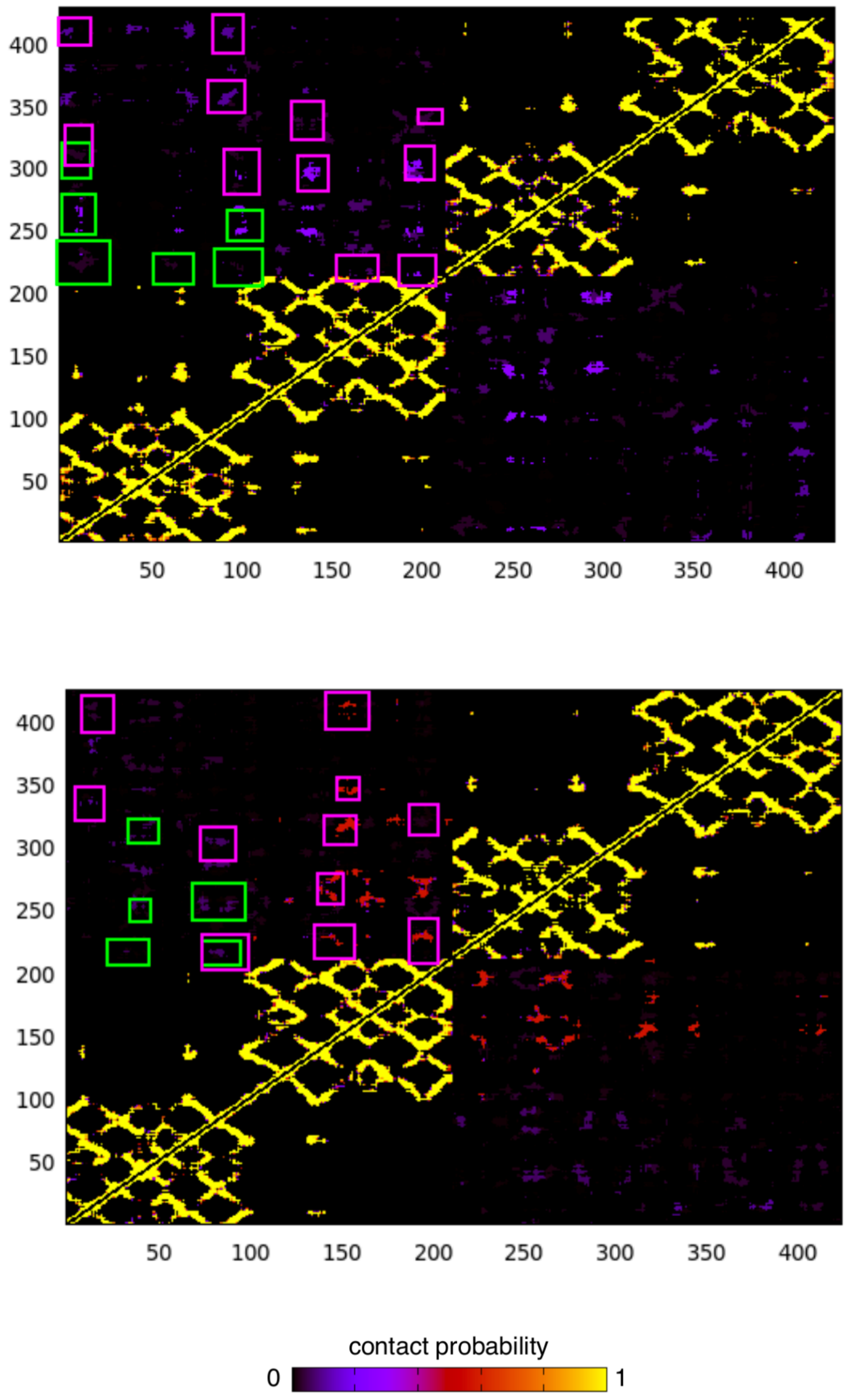

Figure S6: the simulated contact maps of N-cadherin (above) and P-cadherin (below). Green boxes mark contacts present in the strand dimer, purple boxes those present in X-dimers.

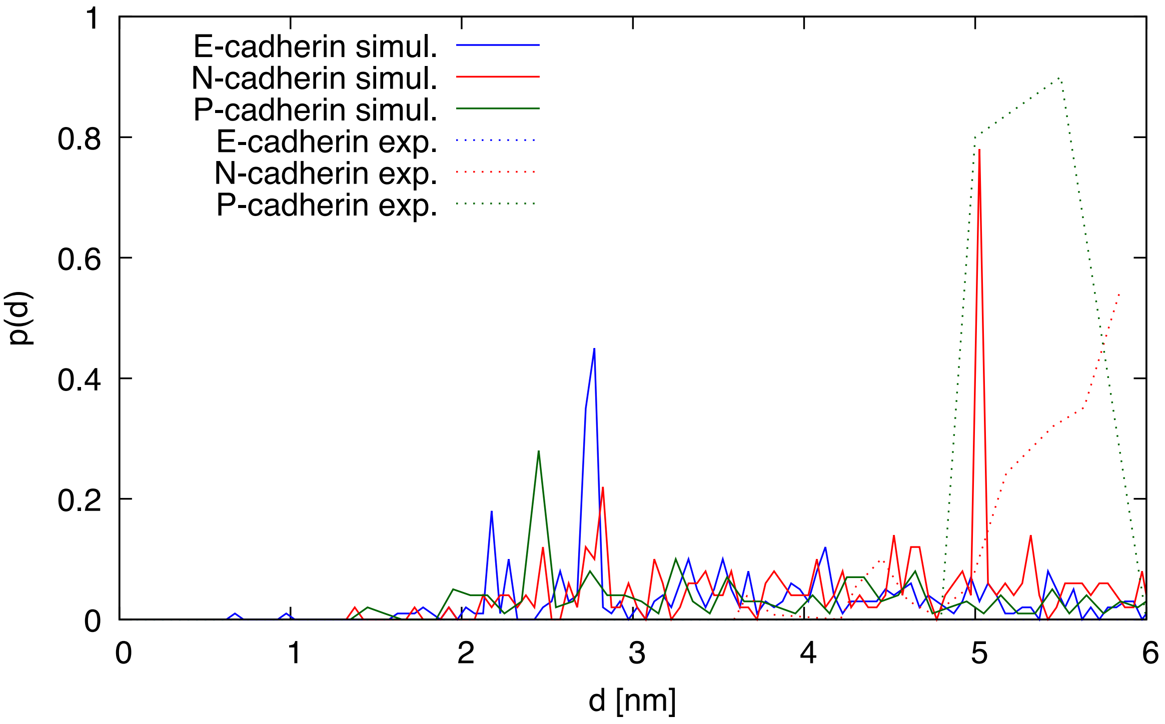

Figure S7: comparison of the simulated distribution of distances between residues 135 with the results of DEER experiments.
